# Timing and locations of reef fish spawning off the southeastern United States

**DOI:** 10.1371/journal.pone.0172968

**Published:** 2017-03-06

**Authors:** Nicholas A. Farmer, William D. Heyman, Mandy Karnauskas, Shinichi Kobara, Tracey I. Smart, Joseph C. Ballenger, Marcel J. M. Reichert, David M. Wyanski, Michelle S. Tishler, Kenyon C. Lindeman, Susan K. Lowerre-Barbieri, Theodore S. Switzer, Justin J. Solomon, Kyle McCain, Mark Marhefka, George R. Sedberry

**Affiliations:** 1 NOAA/National Marine Fisheries Service, Southeast Regional Office, St. Petersburg, Florida, United States of America; 2 LGL Ecological Research Associates, Inc., Bryan, Texas, United States of America; 3 NOAA/National Marine Fisheries Service, Southeast Fishery Science Center, Miami, Florida, United States of America; 4 Department of Oceanography, Texas A&M University, College Station, Texas, United States of America; 5 South Carolina Department of Natural Resources, Marine Resources Research Institute, Charleston, South Carolina, United States of America; 6 Meadows Ecological, Inc., Indialantic, Florida, United States of America; 7 Florida Institute of Technology, Melbourne, Florida, United States of America; 8 Florida Fish and Wildlife Conservation Commission, Florida Fish and Wildlife Research Institute, St. Petersburg, Florida, United States of America; 9 Florida Fish and Wildlife Conservation Commission, Florida Fish and Wildlife Research Institute, Jacksonville Field Laboratory, Jacksonville, Florida, United States of America; 10 Abundant Seafood, Charleston, South Carolina, United States of America; 11 NOAA Office of National Marine Sanctuaries, Savannah, Georgia, United States of America; Department of Agriculture and Water Resources, AUSTRALIA

## Abstract

Managed reef fish in the Atlantic Ocean of the southeastern United States (SEUS) support a multi-billion dollar industry. There is a broad interest in locating and protecting spawning fish from harvest, to enhance productivity and reduce the potential for overfishing. We assessed spatiotemporal cues for spawning for six species from four reef fish families, using data on individual spawning condition collected by over three decades of regional fishery-independent reef fish surveys, combined with a series of predictors derived from bathymetric features. We quantified the size of spawning areas used by reef fish across many years and identified several multispecies spawning locations. We quantitatively identified cues for peak spawning and generated predictive maps for Gray Triggerfish (*Balistes capriscus*), White Grunt (*Haemulon plumierii*), Red Snapper (*Lutjanus campechanus*), Vermilion Snapper (*Rhomboplites aurorubens*), Black Sea Bass (*Centropristis striata*), and Scamp (*Mycteroperca phenax*). For example, Red Snapper peak spawning was predicted in 24.7–29.0°C water prior to the new moon at locations with high curvature in the 24–30 m depth range off northeast Florida during June and July. External validation using scientific and fishery-dependent data collections strongly supported the predictive utility of our models. We identified locations where reconfiguration or expansion of existing marine protected areas would protect spawning reef fish. We recommend increased sampling off southern Florida (south of 27° N), during winter months, and in high-relief, high current habitats to improve our understanding of timing and location of reef fish spawning off the southeastern United States.

## Introduction

The South Atlantic Fishery Management Council (Council) manages 50 Atlantic Ocean reef fish stocks (e.g., snappers, groupers, porgies, grunts, tilefishes) over nearly 500,000 km^2^ of federal waters off the southeastern United States (SEUS) from Florida to North Carolina. These stocks provide billions of dollars to coastal communities through commercial and recreational fisheries and tourism [1]. Management of reef fish is commonly based on maximizing reproductive potential, expressed as spawning stock biomass or total egg production [2]; however, in many marine species, the location and timing of spawning may be more important to reproductive success [3]. Recognizing that spawning areas are productivity hotspots where small investments in research and management can lead to large benefits for fisheries and conservation [4], the Council is currently contemplating a variety of management measures designed to protect spawning reef fish [5,6].

Reef fish exhibit a great diversity of spawning strategies [7]. Resident spawners have protracted spawning within their home range, whereas transient spawners migrate relatively large distances to spawn in aggregations during only a portion of the year [8]. More recently, this distinction has been illustrated as a suite of non-linear continua dependent on distance migrated to spawn, number of individuals aggregated to spawn, duration of spawning, and other variables [9,10]. Choat [11] analyzed factors that determine spawning behaviors and found that body size is the most important predictor. Specifically, species with large (> 40 cm mean maximum fork length) bodies (regardless of age, diet, or lineage) were likely to spawn in transient aggregations.

Species that are long-lived, migrate long distances, and form dense transient aggregations are extremely vulnerable to overfishing [12]. Once discovered by fishers, transient aggregation sites may be rapidly extirpated [12–14]. Overfishing transient aggregations may lead to sperm limitation in protogynous hermaphrodites [15] and removal of gravid individuals prior to spawning. This has resulted in the elimination of spawning aggregations for some species [16]. Once eliminated, spawning aggregation sites may not re-form, or they may form in less-desirable locations with respect to larval survival, leading to declines in the exploited stock [17–19]. Protection of a site prior to extirpation may lead to substantial increases in spawning activity [20] and rebuilding of the exploited stock [21,22]. Spawning sites are commonly used by multiple species [7,17–19,23–25]; thus, identification and protection of a site used by one species may directly benefit other species and serve as an effective precautionary approach to management [26].

The precise timing of spawning may be synchronized to lunar, solar, diel, and/or tidal cycles [27–30]. Studies in the Caribbean have suggested reef geomorphology may be a key determinant in the selection of reef fish spawning locations [25,31–34]. Spawning locations in Belize and the Cayman Islands were generally near the inflection points of convex-shaped reefs in 20–40 m water depth, adjacent to sharp shelf edges where water depths drop several hundred meters [25]. These features appear to be common to other spawning locations in Cuba [35], Florida [36], and Mexico [37], as they endure for thousands of years and create unique current patterns that promote either local larval retention or long-distance dispersal depending on currents at the time of spawning [38].

The objectives of this study were to: 1) synthesize what is known about timing of spawning for managed reef fish stocks in the Atlantic Ocean off the SEUS, 2) quantitatively test what variables are predictive of spawning activity, 3) generate spatial predictions of spawning locations, 4) validate predicted spawning locations based on the ecological knowledge of local fishermen and scientific field studies, and 5) suggest needed data and methods for prediction and verification of the locations of spawning aggregations. Our results may help the Council update information on periods when spawning activity is highest and delineate the appropriate locations and spatiotemporal extent for marine protected areas (MPAs) to protect spawning fish.

## Materials and methods

### Data sources

#### Histological samples

The primary source of data for this project was the Southeast Reef Fish Survey (SERFS) database. SERFS is a long-term fishery-independent monitoring program targeting reef fish species of the SEUS Atlantic Ocean. We used SERFS data from the Marine Resources Monitoring, Assessment and Prediction (MARMAP) program (1990–2013), the Southeast Area Monitoring and Assessment Program, South Atlantic region (SEAMAP-SA) Reef Fish Complement (2009), and the Southeast Fishery Independent Survey (SEFIS; 2010–2013). By using consistent gears (e.g., chevron traps and bottom longlines) and methodologies over time, SERFS has facilitated long-term examinations of changes in relative abundance, species distribution, and life history traits for a variety of fish species. Throughout the survey history, the SERFS has collected biological data for investigation of population demographics (e.g., length, age, and sex composition) and life history traits (e.g., growth rates, age/length-at-maturity/transition, spawning season).

**Fig 1:**
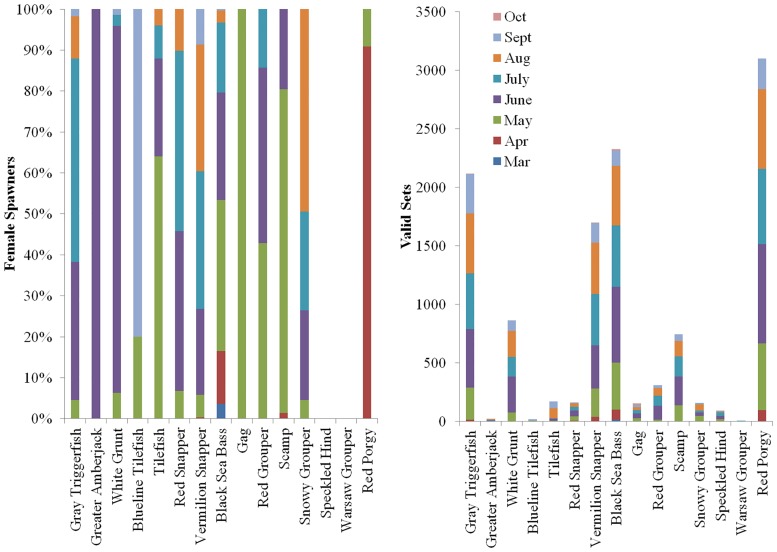
Spawning condition females and valid sets by month. Percentage of SERFS samples of females within 48 hours of spawning (*left*) and number of sets (*right*) where a histological sample was taken, by species and sampling month.

Fishery-independent SERFS data collection efforts were concentrated between May and September, though some samples were collected from all months from March through October (Fig 1). Periodically, the MARMAP program has supplemented SERFS fishery-independent biological data via targeted fishery-dependent sampling of fish wholesalers for particular species to obtain biological samples from species that were caught infrequently by monitoring gears and/or collect biological samples from months and/or seasons of the year outside the routine fishery-independent monitoring season. Such samples were used to determine reproductive life history characteristics of particular species, including spawning seasonality and spawning location information.

In 2012, the Florida Fish and Wildlife Commission (FWC) conducted a stratified random sampling study for Red Snapper from 28° 00’ N (Melbourne, Florida) to 30° 45’ N [39]. Each month, 12 inshore (i.e., <30 m depth) and 20 offshore (i.e., depths of 30–100 m) sites were sampled using standardized fishing methods. Three sampling techniques were used (passively fished vertical and horizontal bottom longlines and an actively fished repetitive timed drop method using Elec-tra-mate gear). Histological indicators were used to assess reproductive state and phase for fish captured during sampling consistent with the definitions below [40,41]. This sampling program also obtained bycatch collections of spawning condition Black Sea Bass and Vermilion Snapper.

For three sites in the South Atlantic the authors attempted field validation of predicted spawning areas, via cooperation with commercial fishermen. Field validation techniques included video monitoring using Go-Pro^™^ drop cameras, catch-per-unit-effort (CPUE) monitoring and collection of biological samples at predicted spawning areas. Histological analysis from these samples was conducted by MARMAP staff and was used to verify spawning condition.

#### Spawning season

Seasonal and lunar cues to spawning activity for reef fish species were compiled from SERFS samples and supplemented with information from peer-reviewed literature, especially stock assessment reports [42]. Timing of peak spawning was noted as well as duration of spawning season.

#### Bathymetric categorization

A complete bathymetric layer for the SEUS was developed within a geographic information system (GIS) from the National Oceanographic and Atmospheric Administration’s (NOAA) Coastal Relief Model (CRM: www.ngdc.noaa.gov/mgg/coastal/startcrm.htm). The CRM provides a comprehensive three arc-second (approximately 90 m) resolution view of the U.S. coastal zone, integrating offshore bathymetry with land topography. The CRM was assimilated from numerous bathymetric sources including U.S. National Ocean Service Hydrographic Database, the U.S. Geological Survey (USGS), the Monterey Bay Aquarium Research Institute, the U.S. Army Corps of Engineers, the International Bathymetric Chart of the Caribbean Sea, the Gulf of Mexico project, and various academic institutions. Topographic data are from the USGS and the Shuttle Radar Topography Mission (SRTM).

Additional high-resolution (3–50 m) multi-beam (MB) bathymetric layers were assimilated from NOAA, SEFIS, USGS, the U.S. Navy, and the National Centers for Coastal Ocean Science (NCCOS: https://products.coastalscience.noaa.gov/collections/benthic/e49s_atlantic/). The MB bathymetric layer covered relatively small areas in and around existing and proposed SEUS MPAs.

The CRM and MB bathymetric layers were categorized according to broad-scale and fine-scale bathymetric positioning index (BS-BPI and FS-BPI), slope, aspect, and curvature. BPI is a second order derivative of the surface, which defines the elevation at locations with reference to the overall landscape. A BPI value less than zero denotes a valley, a BPI equal to zero denotes a flat area, and a BPI greater than zero denotes a ridge. BS-BPI and FS-BPI were computed using Benthic Terrain Modeler [43] with an inner annulus cell size of 1 (i.e., 90 m) and outer annulus sizes of 18 (approximately 1 arc-minute at 30° N) and 9 cells, respectively.

### Definitions

#### Spawning areas

We use the term “spawning areas” to denote locations where individual species of fish actively spawn; these areas may represent the year-round habitat of an individual fish, or may represent specific areas to which fish move for the express purpose of spawning. Spawning areas may contain reproductively active fish engaged in resident, simple migratory, or transient aggregation spawning following definitions in [8,10,44].

Fish spawning in an area can be verified directly in one of two ways. First, actual spawning events (e.g., gamete release) can be recorded using photos or video, which is generally accomplished using scuba in the tropics [8,45,46]. Second, the reproductive phases of fishes (including spawning) can be documented through observations of gonadal tissues. Several indirect methods have also been used traditionally to suggest the presence of spawning aggregations. These include anecdotal evidence from fishermen, photo/video evidence of courtship behaviors or colorations, or high CPUE at the site including catch of some fishes with large, late development stage gonads. Photo or video documentation of courtship coloration or behaviors, swollen or distended abdomens, recent bite marks, or a species density three times the normal density at the site can also provide indirect evidence for the presence of spawning aggregations [8,45,46]. Most of these techniques were developed for use in relatively shallow (25–60 m) shelf-edge tropical waters [47], but can be adapted to the deeper, sometimes turbid waters of the SEUS [48].

In addition to information from fishery-independent surveys and collaborative research projects with fishermen, the timing of spawning (season and lunar phase) for various species has been published in papers and reports (e.g., stock assessment reports). Experienced fishermen are an important source of information on spawning areas and times based on their extensive time at sea [7]. Indeed, multispecies spawning sites have been identified by fishermen in Florida [20,44], Cuba [35], Belize [23,38], the Cayman Islands [24,49], the west Florida shelf [50,51] Brazil [52], and the SEUS [5,44]. Summaries of existing information from fishermen about the timing and location of spawning aggregation areas are important resources [53,54]. Finally, bathymetric data and maps can be used to identify prominent shelf-edge features that may serve as aggregation sites [47].

#### Spawning condition

All reef fish species with sufficient data were evaluated, providing a representative variety of sizes, families, and life histories. Spawning females were defined as mature females with histological evidence of oocyte maturation (nucleus migration through hydration) and/or post-ovulatory follicles in gonadal tissue, indicating the fish was captured within 48 hours of a spawning event [55]. The duration of oocyte maturation through post-ovulatory follicles is influenced by water temperature [56–58]. Based on the water temperatures in the study area, discernable follicles in histological sections were expected until approximately 24–48 hr after spawning.

### Data analyses

#### Multispecies and multi-year use of spawning locations

Multispecies spawning areas were defined as areas where a single collection retrieved spawning condition females of two or more species. Multi-year spawning areas were defined as locations where spawning females of a single species were collected in two or more sampling years from 1990–2013. Characteristics of multi-year spawning areas were determined through visual inspection of underlying bathymetry and distance from the edge of the continental shelf (i.e., shelf-edge). The sizes of these locations were determined using minimum convex polygon methods implemented in Geospatial Modeling Environment [59]. Because it was difficult to quantitatively determine the edges of multi-year spawning areas relative to the underlying sampling scheme, estimates are presented as approximations rounded to the nearest square kilometer.

#### Model selection and validation

Spawning timing and location were modeled, by species, in a binary logistic regression framework for species with sample sizes sufficient to achieve model convergence (i.e., n>90). The logistic regression modeled the probability of detecting a female of a given species in spawning condition versus a female in non-spawning condition, as a function of gear, habitat, latitude, year, month, lunar phase, depth, temperature, and bathymetric features (Table 1). We also tested for interaction factors between latitude and depth, latitude and month, and latitude and temperature, as some species may have different peak spawning times in different areas.

**Table 1:** Input variables considered in logistic regression model.

Variable	Description	Treatment	Coverage
MAXBCURVE	Maximum curvature value from high-resolution bathymetry	Categorical (binned)	Limited
MEANBCURVE	Mean curvature value from high-resolution bathymetry	Categorical (binned)	Limited
MAXBASPECT	Maximum aspect value from high-resolution bathymetry	Categorical (binned)	Limited
MEANBASPEC	Mean aspect value from high-resolution bathymetry	Categorical (binned)	Limited
MAXBSLOPE	Maximum slope value from high-resolution bathymetry	Categorical (binned)	Limited
MEANBSLOPE	Mean slope value from high-resolution bathymetry	Categorical (binned)	Limited
MAXBBSBPI	Maximum broad-scale (18 cell) BPI value from high-resolution bathymetry	Categorical (binned)	Limited
MEANBBSBPI	Mean broad-scale (18-cell) BPI value from high-resolution bathymetry	Categorical (binned)	Limited
MAXBFSBPI	Maximum fine-scale (9 cell) BPI value from high-resolution bathymetry	Categorical (binned)	Limited
MEANBFSBPI	Mean fine-scale (9-cell) BPI value from high-resolution bathymetry	Categorical (binned)	Limited
MAXCBSBPI	Maximum broad-scale (18 cell) BPI value from coastal relief model bathymetry	Categorical (binned)	Comprehensive
MEANCBSBPI	Mean broad-scale (18-cell) BPI value from coastal relief model bathymetry	Categorical (binned)	Comprehensive
MAXCFSBPI	Maximum fine-scale (9 cell) BPI value from coastal relief model bathymetry	Categorical (binned)	Comprehensive
MEANCFSBPI	Mean fine-scale (9-cell) BPI value from coastal relief model bathymetry	Categorical (binned)	Comprehensive
MAXCASPECT	Maximum aspect value from coastal relief model bathymetry	Categorical (binned)	Comprehensive
MEANCASPEC	Mean aspect value from coastal relief model bathymetry	Categorical (binned)	Comprehensive
MAXCCURVE	Maximum curvature value from coastal relief model bathymetry	Categorical (binned)	Comprehensive
MEANCCURVE	Mean curvature value from coastal relief model bathymetry	Categorical (binned)	Comprehensive
MAXCSLOPE	Maximum slope value from coastal relief model bathymetry	Categorical (binned)	Comprehensive
MEANCSLOPE	Mean slope value from coastal relief model bathymetry	Categorical (binned)	Comprehensive
Gear_ID	Sampling gear	Categorical	All records
Month	Month	Categorical	All records
Year	Year	Categorical	All records
Latitude	Latitude for gear set (1° bins from 30.5°-34.5°)	Categorical (binned)	All records
Depth	Depth of gear set (20 m bins from 10–70 m)	Categorical (binned)	All records
Temperature	Temperature at depth of gear deployment	Continuous	Some errors
Lunar3	Lunar luminosity expressed as an approximated continuous wavelet function based on NASA archived data on lunar phase (downloaded from: http://eclipse.gsfc.nasa.gov/phase/phases1901.html) with waxing moons as positive values, waning moons as negative, rounded to the nearest quarter moon.	Continuous and Categorical (binned)	All records
Habitat	Habitat type (hardbottom, possible hardbottom, not hardbottom, or unknown) from 1-degree resolution SEAMAP-1199 grid	Categorical	Limited

The model was fit using all gear deployments where individuals were histologically-examined and categorized with regards to their gender and spawning condition (Table 2). We tested for gear effects to determine whether there was evidence of one gear preferentially selecting for spawning condition individuals, and in the absence of any evidence of gear effects we used the full data set with combined gear types. Based on the biology of the study species, using spawning condition females versus non-spawning condition females (as opposed to males or juveniles), is the best model structure for identifying potential spawning areas within the known range of species occurrence (Table 2). Juveniles and males were not considered in the analysis as many species exhibit ontogenetic shifts, and the model effects may have been confounded with ontogenetically driven or sex-specific habitat preferences. Similarly, including sites where zero individuals of the species were observed would have confounded probability of occurrence with probability of spawning and thus was not desirable.

**Table 2:** Number of collections from MARMAP/SERFS and FWC fishery-independent data with histological sampling (Samples), with number of sets containing females within 48 hours of spawning (Female Spawners), number of sets containing females and males within 48 hours of spawning (All Spawners), number of sets with three or more histologically-sampled fish for the species (Valid Sets), and number Valid Sets within the high-resolution multibeam bathymetry (MB Valid Sets).

Common Name	Scientific Name	Family	Samples	Female Spawners	Mature Non-Spawning Females	Valid Sets	MB Valid Sets
Gray Triggerfish	*Balistes capriscus*	Balistidae	2114	122	1400	799	212
*Greater Amberjack	*Seriola dumerili*	Carangidae	20	3	4	1	0
White Grunt	*Haemulon plumierii*	Haemulidae	861	97	536	375	37
Red Snapper	*Lutjanus campechanus*	Lutjanidae	421	159	180	158	8
Vermilion Snapper	*Rhomboplites aurorubens*	Lutjanidae	1697	1124	949	878	159
*Blueline Tilefish	*Caulolatilus microps*	Malacanthidae	18	8	1	2	0
*Tilefish	*Lopholatilus chamaeleonticeps*	Malacanthidae	171	12	73	87	1
Black Sea Bass	*Centropristis striata*	Serranidae	2324	338	1444	1499	10
*Gag	*Mycteroperca microlepis*	Serranidae	154	1	70	3	1
*Red Grouper	*Epinephelus morio*	Serranidae	308	6	143	70	0
Scamp	*Mycteroperca phenax*	Serranidae	743	105	494	143	94
*Snowy Grouper	*Hyporthodus niveatus*	Serranidae	156	48	55	56	5
*Speckled Hind	*Epinephelus drummondhayi*	Serranidae	93	0	10	4	1
*Warsaw Grouper	*Hyporthodus nigritus*	Serranidae	5	0	0	0	0
*Red Porgy	*Pagrus pagrus*	Sparidae	3098	17	1840	1710	361

Species with names preceded by an asterisk had insufficient samples to be statistically modeled.

We modeled the logistic response in a mixed-model framework where year was treated as a random effect, and all other effects were fixed. Treating year as a random effect allowed us to control for time-varying processes which could potentially affect the ratio of spawning to non-spawning females in a sample, such as annual differences in sampling method (e.g., binning vs. random sampling), size- or age-selective fishing pressure, or recruitment pulses. Month was treated as a factor; other predictor variables were binned by quantiles and also treated as factors to avoid imposing linear relationships between factors and spawning probability. Factor data bins with no observations were removed. Bathymetric variables tested were fine-scale and broad-scale BPI, slope, curvature, and aspect. Mean and maximum values for bathymetric features were statistically summarized for each sample site using a 381.8 m buffer (i.e., the hypotenuse of a 3x3 grid of the CRM’s 90 m cells). Bathymetric features for the MB bathymetry were summarized using a 127 m buffer. To increase statistical power and geographic coverage, the FWC and SERFS data were combined when analyzing Red Snapper. No gear effects were found to be significant.

Logistic regression analysis using a logit-linked generalized linear mixed model was implemented using R version 3.2.3, package *lme4*, function *glmer* [60]. Generalized additive models (GAMs) may have provided better smoothing; however, GAMs and generalized linear models (GLMs) make similar predictions within areas of high sampling [61], and GAMs may overfit the data, especially when considering many variables of unknown importance, resulting in less useful predictions beyond the sampling domain [62]. As our goal was to make predictions for the entire Council jurisdiction, we opted for the simpler GLM approach. GLMs were developed using a two-stage approach. First, models were fit to the suite of non-bathymetric variables that have been reported to affect spawning activity (month, temperature, latitude, depth, lunar phase). Variables with correlations >60% were not included in the same model, to avoid multicollinearity. Once a model was selected based on these factors as described below, the suite of bathymetric variables were tested for inclusion; this was done in a separate stage because a large suite of bathymetric variables was considered, and there was potential for spurious relationships. In the first stage, models were fit in a forward stepwise fashion, testing the suite of non-bathymetric variables (Table 1), and a subset of potential models using these factors was identified based on lowest AIC [63].

Model performance was internally tested using a 10-fold cross-validation procedure in which the data were split into training and testing sets, and a model was fit to the training set and subsequently tested on the “unseen” testing set [64]. Using a receiver operating characteristic (ROC) curve (R ‘pROC’ library; [65]) for each of the subset of AIC-selected models, we calculated the threshold at which the proportion of correctly classified positive observations plus the proportion of correctly classified negative observations are maximized.

Using the parameters defined by each model, as well as the threshold defined by the ROC curve for each model using the training set, we then made predictions for the testing set. The predictive utility of models was rated based on the ROC area-under-the-curve (AUC) as ‘excellent’ (AUC = 90–100%), ‘good’ (80–89%), ‘fair’ (70–79%), and ‘poor’ (<70%). Once the best model containing all or a subset of these factors was defined, based on cross-validation output and lowest AIC, bathymetric variables were tested to see if their inclusion lowered the model AIC. If bathymetric variable inclusion lowered model AIC and improved predictive utility, based on cross-validation as described above, the variable was retained.

Because of the large number of bathymetric variables tested, some of which had limited contrast, we carried out an additional randomization test to determine the likelihood that any significant associations with bathymetric variables were spurious. This test involved: 1) retaining all non-bathymetric variables in the model at their original values and randomizing each of the variables independently such that they were random variables retaining their original distributions, 2) refitting the model with each randomized bathymetric variable individually, and 3) identifying the randomized bathymetric variable that led to the largest reduction in AIC, and 4) calculating the percent deviance explained by the randomized variable. This process was repeated 500 times to determine the percentage of instances where inclusion of a randomized bathymetric variable explained more deviance in the data than the true selected bathymetric variable. This percentage is then equivalent to the probability that the inclusion of a bathymetric variable in the final model was spurious.

#### Independent validation of predicted spawning locations

Maps of probability of a spawning condition female at time of peak spawning were generated, by species, for a grid covering SEUS waters from 25° N to 35° N to 200 m depth. The resolution of the grid corresponded to the resolution used to compute CRM bathymetric statistics (270 m × 270 m). Model predictions were based on the given latitude, depth, and bathymetric features for each grid cell, with month, temperature, and/or lunar phase effects fixed at their peak values for the prediction. Predictive grid values were converted to Z-scores, by species.

We gathered independent validation data for predicted spawning areas from: 1) interviews with fishermen who had knowledge of spawning times and locations based on experience, 2) collaborative field research, and 3) overlays of supplemental fishery-independent and fishery-dependent data sources. Tishler-Meadows [66] collected confidential information on spawning areas from fisher interviews. LGL Ecological Research Associates, Inc. (LGL-ERE) conducted a cooperative field research program with commercial fishermen in 2014–2015 that provided histological samples from locations off South Carolina and North Carolina [67]. FWC (2012–2013) collected fishery-independent bycatch and MARMAP (1974–2014) collected fishery-dependent samples that were histologically evaluated. The FWC bycatch and MARMAP fishery-dependent samples could not be used in a predictive modeling framework because they were not collected through a structured sampling program for the stock that included areas where no spawning condition individuals were observed (e.g., the zeroes); however, both were suitable for external validation of model predictions. For independent validation comparisons, positive Z-scores underlying independent validation sites were interpreted as supportive of the model predictions.

## Results

### Multispecies and multi-year use of spawning locations

**Fig 2:**
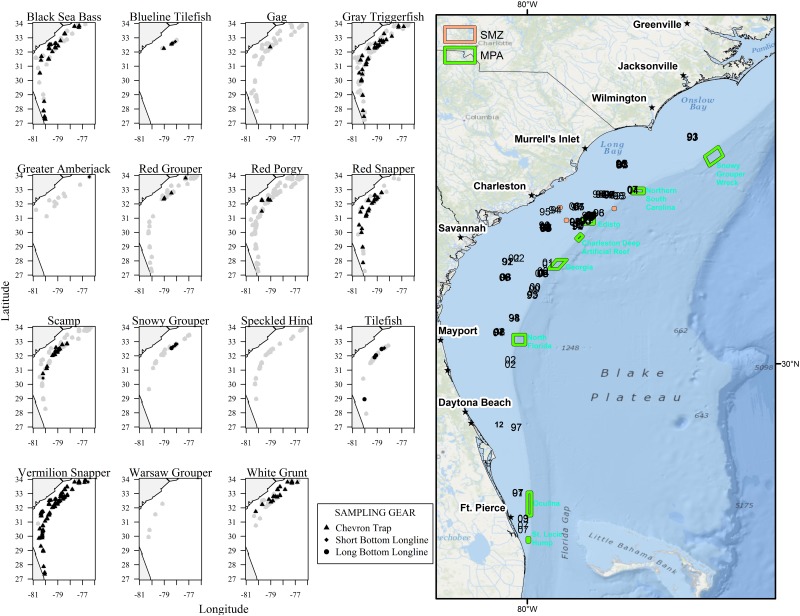
Fishery-independent sampling and multispecies spawning locations. On left, fishery-independent samples of female fish within 48 hours of spawning, by species. Gray shapes denote histological samples, black shapes denote collections of spawning condition females, with triangles denoting chevron traps, diamonds denoting short bottom longline, and circles denoting long bottom longline. On right, sites where females of multiple species have been captured in spawning location at the same time (labeled by collection year). Green boxes denote no-take marine protected areas. Basemap courtesy ESRI and National Park Service.

Spawning records were available for 15 species and varied by latitude and environmental factors (Fig 2, Table 3). No spawning Speckled Hind *Epinephelus drummondhayi* or Warsaw Grouper *Hyporthodus nigritus* and few Gag *Mycteroperca microlepis*, Greater Amberjack *Seriola dumerili*, and Red Grouper *Epinephelus morio* were encountered by fishery-independent SERFS sampling.

**Table 3:** Summary statistics for water depth (m), salinity (ppt), and temperature (°C) during SERFS observations of spawning condition females.

Common name	Scientific name		N	Valid	Mean	SD	Min	Max
Gray Triggerfish	*Balistes capriscus*	Depth	122	122	44.8	9.5	20	75
		Salinity	122	121	36.3	0.2	35	37
		Temp	122	121	22.7	2.2	15	27
White Grunt	*Haemulon plumierii*	Depth	97	97	33.3	8.8	22	52
		Salinity	97	97	36.1	0.3	35	37
		Temp	97	97	23.7	1.9	18	27
Blueline Tilefish	*Caulolatilus microps*	Depth	8	8	181.9	33.8	100	205
		Salinity	8	7	36.1	0.0	36	36
		Temp	8	7	16.0	0.3	15	16
Tilefish	*Lopholatilus chamaeleonticeps*	Depth	12	12	204.5	16.6	188	240
		Salinity	12	11	35.7	0.3	35	36
		Temp	12	11	12.8	2.1	10	16
Red Snapper	*Lutjanus campechanus*	Depth	40	40	43.2	11.6	23	66
		Salinity	40	40	36.2	0.3	35	37
		Temp	40	40	22.6	2.5	17	28
Vermilion Snapper	*Rhomboplites aurorubens*	Depth	1124	1124	36.9	12.4	18	104
		Salinity	1124	1085	36.1	2.1	1	39
		Temp	1124	1085	23.4	2.5	13	28
Black Sea Bass	*Centropristis striata*	Depth	338	338	25.4	9.1	15	66
		Salinity	338	335	35.7	0.8	34	40
		Temp	338	335	19.5	2.6	11	27
Red Grouper	*Epinephelus morio*	Depth	6	6	50.5	14.5	28	73
		Salinity	6	6	36.3	0.1	36	37
		Temp	6	6	20.5	2.4	17	24
Scamp	*Mycteroperca phenax*	Depth	105	105	52.1	13.2	32	101
		Salinity	105	100	36.3	0.1	36	37
		Temp	105	100	21.0	2.1	16	27
Snowy Grouper	*Hyporthodus niveatus*	Depth	48	48	190.0	9.1	175	223
		Salinity	48	29	35.6	0.4	35	36
		Temp	48	29	13.4	2.0	9	15
Red Porgy	*Pagrus pagrus*	Depth	17	17	38.6	18.5	26	93
		Salinity	17	16	36.3	0.3	35	36
		Temp	17	16	16.9	0.9	16	19

N: number of samples with spawning females; Valid: number of samples with water metrics >0; SD: standard deviation.

Multiple species were observed in spawning condition on 128 of 10146 SERFS collections (1.3%). Vermilion Snapper spawning condition females were observed at multispecies spawning areas with Black Sea Bass, Gray Triggerfish, Scamp, Red Snapper, and White Grunt (Table 4). On six of these 128 multispecies sets, females of three different species were captured in spawning condition. These encounters were composed of combinations of Black Sea Bass, Gray Triggerfish, Red Snapper, Scamp, Vermilion Snapper, and White Grunt. Red Snapper were encountered at five of these six events. Multispecies spawning observations were primarily found off Charleston, SC and Savannah, GA within the historical core SERFS sampling domain (Fig 2). Repeated observations of multispecies spawning were noted in many areas, including three sites in the Northern SC MPA and nine sites in the Edisto MPA (Fig 2).

**Table 4:** Number of gear deployments with multispecies observations of spawning females from SERFS.

Stock	Gray Triggerfish	White Grunt	Red Snapper	Vermilion Snapper	Black Sea Bass	Scamp	Red Porgy
Gray Triggerfish		7	6	25	0	0	0
White Grunt	7		2	16	4	3	0
Red Snapper	6	2		12	1	3	0
Vermilion Snapper	25	16	12		40	15	0
Black Sea Bass	0	4	1	40		1	4
Scamp	0	3	3	15	1		1
Red Porgy	0	0	0	0	4	1	

Multispecies spawning areas tended to be located on the shelf-edge or inshore, with the densest cluster of observations in the core SERFS sampling range along the shelf-edge in and around the Edisto and Northern South Carolina MPAs. Additionally, FWC had one record of spawning condition Vermilion Snapper and Black Sea Bass caught together off northern Florida. Of the 128 SERFS collections with observations of multispecies spawning, only two were within 110 m of each other in different years, and both had observations of multispecies spawning in both years sampled. Of the 44 sites with multispecies spawning observations within 1.1 km of another collection, 27 (61%) had repeat observations, including many sites with observations across more than two years.

**Fig 3:**
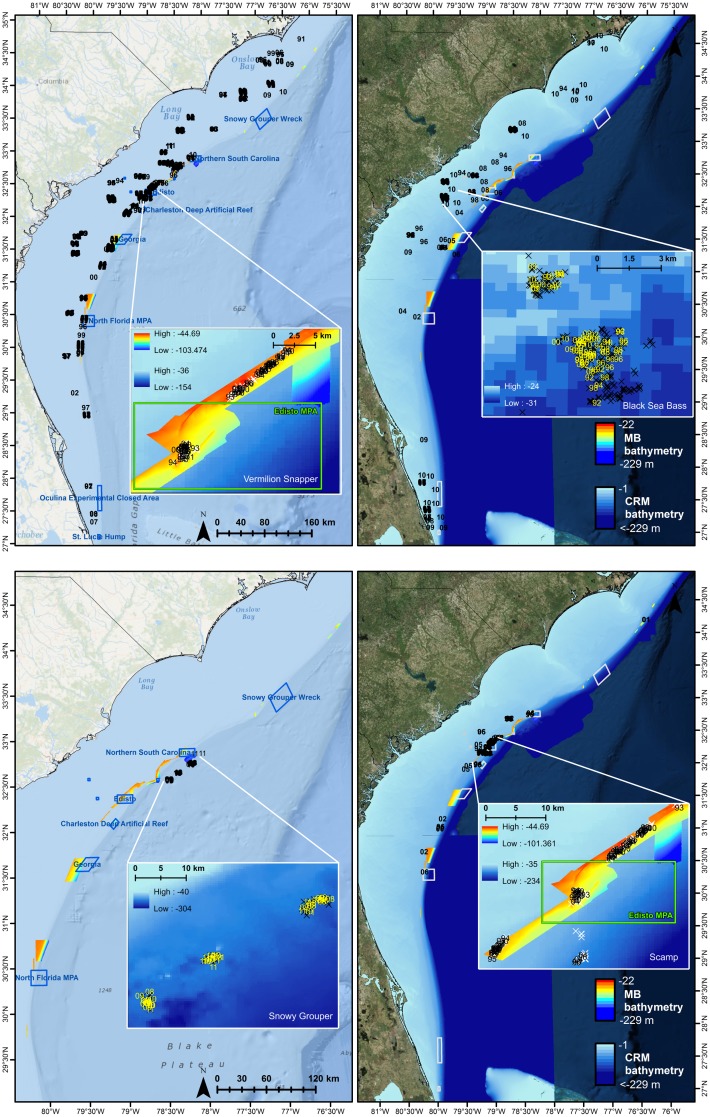
Multi-year observations of spawning. Sites at which spawning condition females were collected in multiple years (two-digit labels) for Vermilion Snapper (top left), Black Sea Bass (top right), Snowy Grouper (bottom left), and Scamp (bottom right) relative to bathymetry, histological sampling locations (Xs), marine protected areas and SMZs (blue/gray boxes).

Most species evaluated appeared to use the same general spawning locations across multiple years (Fig 3, Table 5). Gray Triggerfish were observed over multiple years at 17 areas on or just inshore of the shelf-edge; areas ranged in size from 0–16 km^2^. White Grunt were observed over multiple years at 14 shelf-edge and inshore areas ranging in size from 0–6 km^2^. Red Snapper were observed over multiple years at nine shelf-edge and inshore areas; areas ranged in size from 0.01–5 km^2^. One FWC sampling site overlapped with SERFS sampling, and female Red Snapper were observed in spawning condition at this site in each of the five years sampled. Vermilion Snapper were observed over multiple years at 42 shelf-edge and inshore areas ranging in size from 0.01–69 km^2^ (Fig 3). Despite limited sampling during their peak spawning season, Black Sea Bass were observed over multiple years at 14 shelf reefs and shelf-edge areas ranging in size from 0.1–13 km^2^ (Fig 3). Spawning Scamp were observed at 14 areas, located predominantly along the shelf edge in and around Edisto MPA, at three offshore edges near the continental rise to the south of the Northern South Carolina MPA, and at some offshore pinnacles. Multi-year spawning locations for Scamp ranged in size from 0.2–8 km^2^. Snowy Grouper were observed over multiple years at three areas offshore of the shelf-edge; areas ranged in size from 2–5 km^2^.

**Table 5:** Summary statistics (mean ± standard deviation) for apparent use of multi-year spawning locations. Multi-year spawning location size computed as minimum convex polygon containing all collections within a site.

Stock	Multi-year Spawning Locations	Years Sampled	% Years with Spawning Condition Females	Multi-year Spawning Location Size (km^2^)
Gray Triggerfish	17	9.9 ± 6.2	51% ± 24%	2.7 ± 4.7
White Grunt	14	6.7 ± 4.2	53% ± 27%	1.6 ± 1.8
Red Snapper	9	2.9 ± 1.2	100% ± 0%	1.0 ± 2.0
Vermilion Snapper	42	9.1 ± 6.2	89% ± 16%	6.4 ± 13.6
Black Sea Bass	14	12.8 ± 6.1	51% ± 22%	4.2 ± 4.2
Scamp	11	8.2 ± 4.2	47% ± 19%	1.8 ± 2.5
Snowy Grouper	3	3.3 ± 0.6	100% ± 0%	3.2 ± 1.4

### Timing and location of spawning

A comprehensive literature review revealed protracted spawning seasons for many species (Table 6). Peak spawning was identified for most species, typically based on gonadosomatic index (GSI). For many species, GSI or other spawning indicators (e.g., direct observation of spawning, passive acoustic detections of spawning sounds) peaked between April and August (Table 6). A period of peak spawning was not identified for Speckled Hind or Warsaw Grouper, nor did SERFS fishery-independent sampling observe spawning condition females for these stocks. The core of SERFS fishery-independent sampling occurs from May-September (Fig 1), which overlaps multiple peak spawning months for Gray Triggerfish, White Grunt, Red Snapper, Vermilion Snapper, Scamp off North Carolina, Snowy Grouper, Blueline Tilefish, and Tilefish (Table 6). Winter months were not well-sampled, limiting collections of spawning condition Black Sea Bass, Red Porgy, and Gag.

**Table 6:** Timing of spawning (gray shading) and peak spawning (black shading) for exploited Atlantic Ocean reef fish stocks off the southeastern United States. Months in bold denote core SERFS core fishery-independent sampling months. See S1 Table for references.

**Stock**	Jan	Feb	Mar	Apr	**May**	**Jun**	**Jul**	**Aug**	**Sep**	Oct	Nov	Dec	**Citation**
Gray triggerfish													[10]
Greater amberjack													[7]
White grunt													[14, 17]
Cubera Snapper													WDH, pers. comm.
Red snapper													[17, 18]
Vermilion snapper													[2, 17]
Blueline tilefish													[6]
Tilefish													[4, 17]
Black sea bass													[15, 17]
Gag													[13, 17]
Red grouper													[1]
Scamp (NC)													[12]
Scamp (FL)													[5]
Scamp (29.95–32.95 °N)													[8, 17]
Snowy grouper													[16, 19]
Speckled hind													[20]
Warsaw Grouper													[11, 17]
Red porgy													[3, 17]

**Fig 4:**
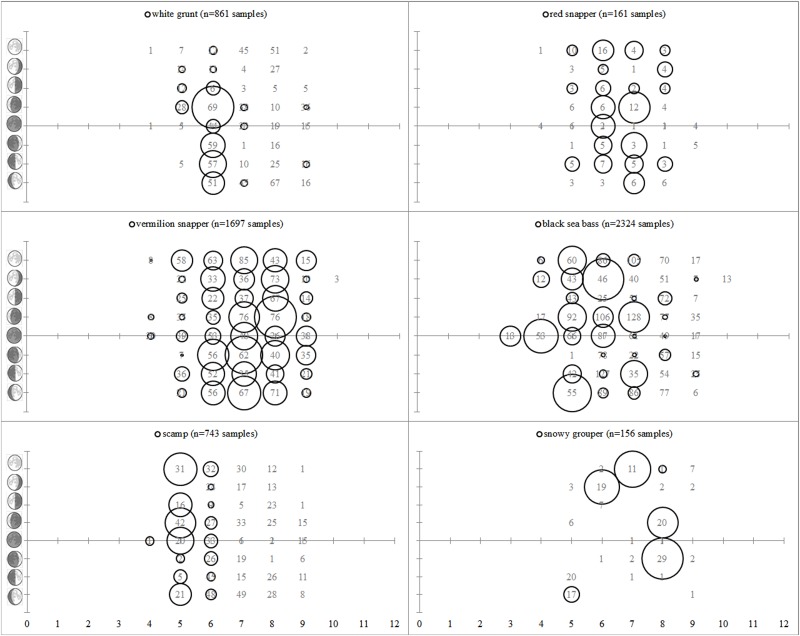
Lunar cycle and spawning. SERFS fishery-independent samples of female fish within 48 hours of spawning (denoted by size of circles; values vary by species) relative to lunar phase and month. Numbers denote histologically examined fish.

Plots of spawning relative to month and lunar phase revealed patterns for several species (Fig 4). Spawning White Grunt were most commonly observed in June during the waxing crescent moon. Spawning Red Snapper were most commonly observed from June-July during the new-waning gibbous moons. Spawning Vermilion Snapper were most commonly observed from June-August during all moons. Spawning Black Sea Bass were most commonly observed from April-July during the waxing crescent, waxing gibbous, and waning gibbous moons. Spawning Scamp were most commonly observed in May during all moon phases. Spawning Snowy Grouper were most commonly observed from June-August during the waning crescent-full moons.

**Fig 5:**
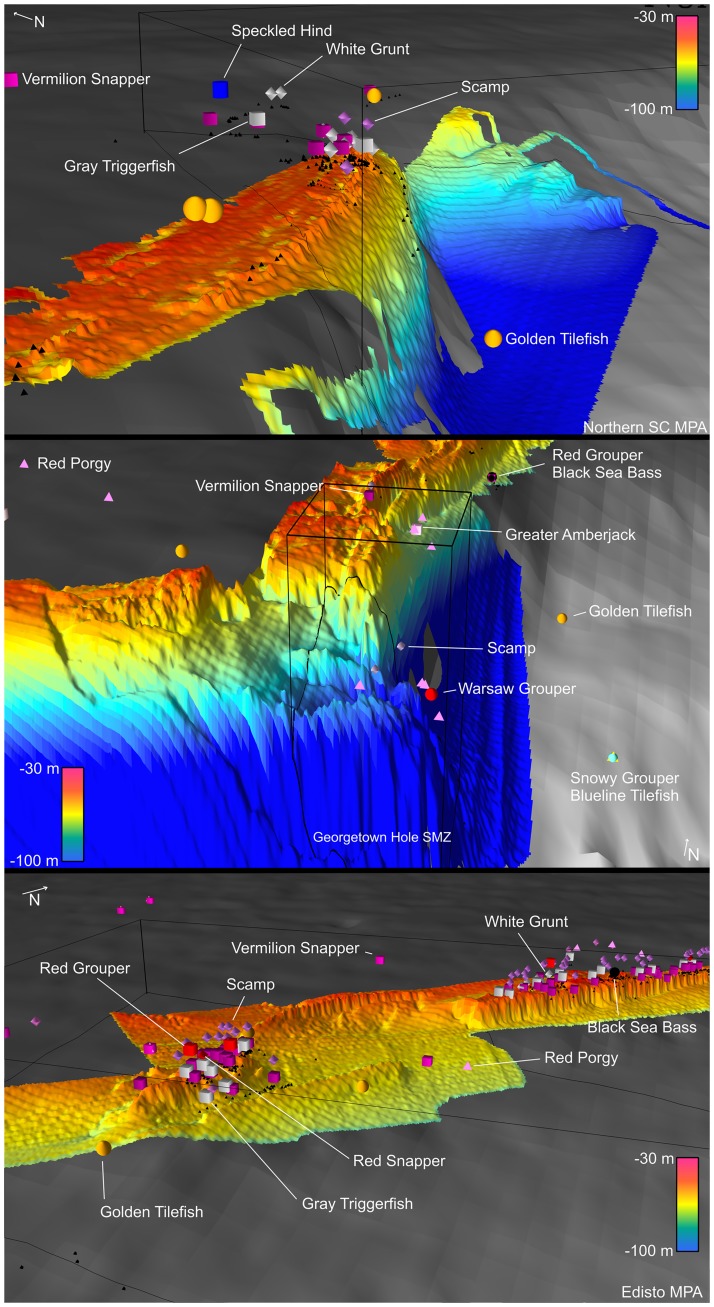
Spawning locations relative to 3D bathymetry. SERFS fishery-independent samples of female fish within 48 hours of spawning, by species, relative to multibeam (MB; rainbow gradient) and Coastal Relief Model (CRM; grayscale gradient) bathymetry near Northern South Carolina MPA (top), Georgetown Hole, SC (middle), and Edisto MPA (bottom). Spawning condition females are shown as floating 3D shapes above SERFS samples (black triangles). Figure created in ArcScene (ESRI, Redlands, CA) using Z-values from MB and CRM with 50-fold vertical exaggeration.

**Fig 6:**
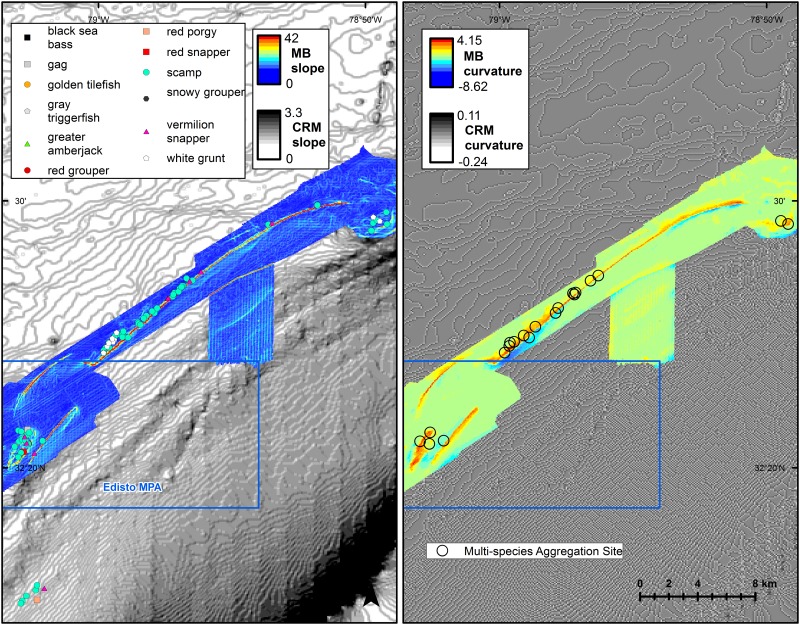
Spawning locations relative to bathymetric slope and curvature. SERFS fishery-independent samples of female fish within 48 hours of spawning, by species, relative to multibeam (MB) and Coastal Relief Model (CRM) bathymetric slope (left) and curvature (right) in area north of Edisto MPA off South Carolina. SERFS histologically-evaluated collection sites are shown as gray Xs in right panel.

Comparison of collection sites of spawning reef fish relative to three-dimensional bathymetry suggested strong associations with high vertical structure for many species (Fig 5, S2 File). Live bottom habitats off SC were well-sampled and spawning areas were most well-defined in this area. Spawning Scamp, Gray Triggerfish, White Grunt, and Vermilion Snapper were captured atop the shelf-edge convex reef crest within the Northern South Carolina MPA (Fig 5: top). Spawning Vermilion Snapper, Scamp, Red Porgy, and Greater Amberjack were captured both along the shelf-edge reef crest north of Georgetown Hole, SC; spawning Blueline Tilefish, Snowy Grouper, and Tilefish were captured in the deeper water at the base of the slope (Fig 5: middle). A spawning Warsaw Grouper was captured by LGL-ERE within the Council’s recently approved spawning Special Management Zone (SMZ) at Georgetown Hole (Fig 5: middle). Spawning Vermilion Snapper, Scamp, Gray Triggerfish, Black Sea Bass, Red Porgy, Red Snapper, and White Grunt were all frequently captured along the shelf-edge reef crest both within and north and south of Edisto MPA (Fig 5: bottom). Areas of high bathymetric slope and curvature appeared to be good predictors of where spawning females and multispecies spawning areas would be located; however, it was difficult to visually assess the confounding effects of sampling effort (Fig 6).

### Model predictions

Sufficient (n>90) fishery-independent collections of spawning condition females were available to fit statistical models for six species: Gray Triggerfish, White Grunt, Red Snapper, Vermilion Snapper, Black Sea Bass, and Scamp (Table 2). Vermilion Snapper was the only stock with sufficient observations of females within the MB bathymetry (n = 179) to test using a 127-m buffer. Regression model fits for timing and location of collections of spawning condition females are presented in tabular format in Table 7 and visually in the S1 File. In the text below, variables are presented in order of descending variability explained.

**Table 7:** Logistic regression model fit statistics for probability of encountering a fish within 48 hours of spawning, with percent deviance explained (i.e. percent variability explained by inclusion of additional variable); cross-validation results for Area Under the Curve (AUC), False Positive Rate (FPR) and False Negative Rate (FNR); and percentage of 500 runs where random variable inclusion in model outperformed model-selected bathymetric variable (‘Random test’). See Methods for additional details.

Stock	Resolution (m)	Total Deviance Explained	AUC	FPR	FNR	Random Test	Percent Deviance Explained							
							Year	Bathymetric Variable	Temp (°C)	Month	Latitude	Depth	Lunar Phase	Interaction
Gray Triggerfish (*Balistes capriscus*)	382	34.35	81.93	24.91	25.17	6.00	11.02	MEANCASPECT (1.91)		10.28	2.02	2.41	2.49	
White Grunt (*Haemulon plumerii*)	382	72.00	90.31	17.27	23.83	100.00	16.55	MEANCFSBPI (1.33)	2.45	25.42	13.63	8.38	10.70	
Vermilion Snapper (*Rhomboplites aurorubens*)	382	30.44	72.85	39.29	32.24	8.33	4.98	MAXCFSBPI (1.60)		<0.01	<0.01	4.16	1.57	Latitude × Month (18.94)
	127	48.24	67.99	48.60	47.60	6.00	19.91	MEANBFSBPI (7.46)	10.75	6.46		2.12	5.71	
Red Snapper* (*Lutjanus campechanus*)	382	46.38	85.15	34.46	15.25	2.00	<0.01	MEANCCURVE (4.91)	3.64	29.65	0.39	0.67	7.71	
Black Sea Bass (*Centropristis striata*)	382	71.67	92.35	17.99	10.16	41.00	5.22	MAXCFSBPI (1.33) MEANCCURVE (1.17)	47.80		10.31	2.67	6.69	
Scamp (Mycteroperca phenax)	382	43.47	82.57	25.30	32.69	64.72	13.03	MEANCFSBPI (2.13)	12.04		9.93	3.44	8.19	

*includes FWC data.

For Gray Triggerfish, 34% of the total variability in the presence of spawning condition females was explained by year, month, lunar phase, depth, latitude, and mean aspect from the CRM bathymetry (Table 7, S1 Fig). Based on AUC, the predictive utility of the model was good. Randomization testing indicated a low risk of incorporating a spurious bathymetric variable.

For White Grunt, 72% of the total variability in the presence of spawning condition females was explained by month, year, latitude, lunar phase, depth, temperature, and mean BPI from the CRM bathymetry (Table 7, S2 Fig). Predictive utility of the model was excellent. Although randomization testing suggested an extremely high risk of incorporating a non-informative bathymetric variable, cross-validation indicated better model performance with the variable included in the final model.

**Fig 7:**
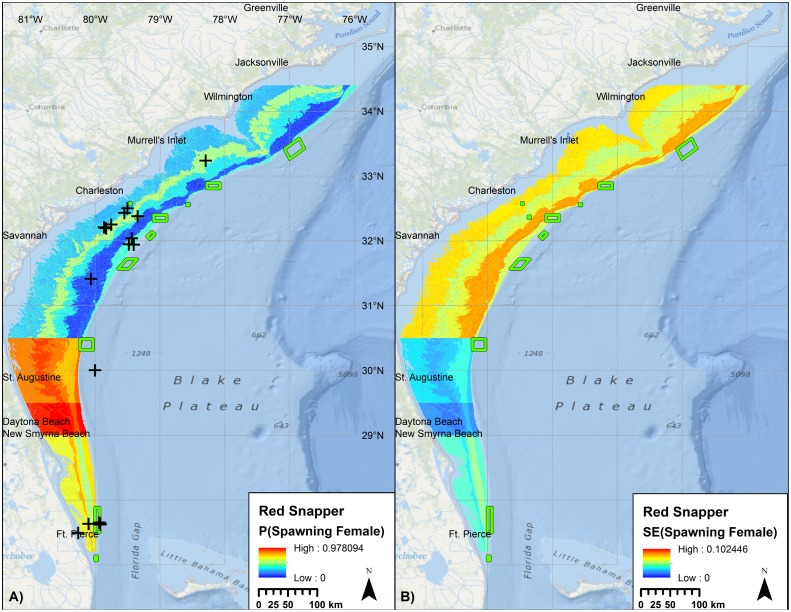
Probability of encountering a spawning condition female Red Snapper. Predicted mean (left) and standard error (right) probabilities of observing spawning condition female Red Snapper at time and conditions of peak spawning, relative to external validation observations (+). Raster color-coding based on percent clip. Green boxes denote no-take marine protected areas and SMZs. Basemap courtesy ESRI Ocean Basemap and partners.

For Red Snapper, 46% of the total variability in the presence of spawning condition females was explained by month, lunar phase, mean curvature from the CRM bathymetry, temperature, depth, latitude, and year (Table 7, Fig 7). Based on AUC, predictive utility of the model was good. Randomization testing indicated a low risk of incorporating a spurious bathymetric variable. Distinct gradients were observed in the predictive map due to quartile binning of latitude.

**Fig 8:**
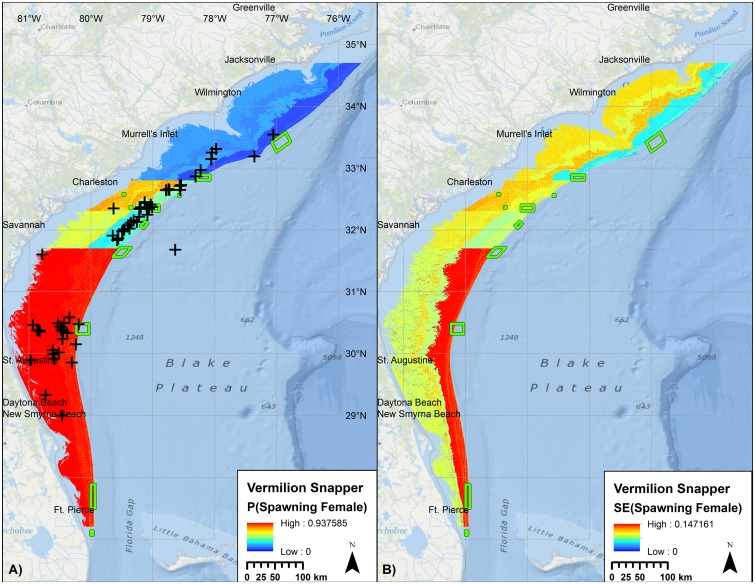
Probability of encountering a spawning condition female Vermilion Snapper. Predicted mean (left) and standard error (right) probabilities of observing spawning condition female Vermilion Snapper at time and conditions of peak spawning, relative to external validation observations (+). Raster color-coding based on 2.5 standard deviations from the mean. Green boxes denote no-take marine protected areas and SMZs. Basemap courtesy ESRI Ocean Basemap and partners.

For Vermilion Snapper throughout the SERFS sampling domain, 30% of the total variability in the presence of spawning condition females was explained by an interaction between latitude and month, year, depth, maximum BPI from the CRM bathymetry, and lunar phase (Table 7, Fig 8). Predictive utility of the model was fair. Randomization testing indicated a relatively low risk of incorporating a spurious bathymetric variable. Distinct gradients were observed in the predictive map due to quartile binning of latitude.

**Fig 9:**
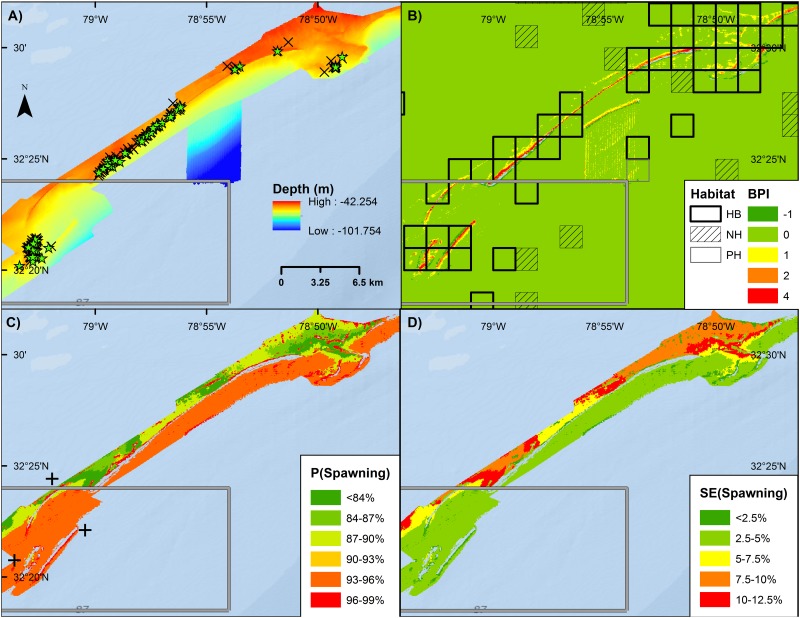
Vermilion Snapper spawning. Maps of Edisto MPA (green) square and surrounding shelf edge showing **A)** Depth from multibeam bathymetry and SERFS samples with spawning condition (stars) and non-spawning condition (Xs) female vermilion snapper, **B)** BPI from Benthic Terrain Modeler and squares denoting habitat type (HB: hardbottom, NH: not hardbottom, PH: potential hardbottom) from SEAMAP-SA, **C)** Model predictions of spawning locations at month and lunar phase of peak spawning and MARMAP fishery-dependent samples of spawning condition female Vermilion Snapper (crosses), and **D)** standard error in model predictions of peak spawning.

For a subset of spawning condition female Vermilion Snapper within the MB bathymetry off South Carolina, 48% of the total variability in the presence of spawning condition females was explained by year, temperature, mean BPI from the MB bathymetry, month, lunar phase, and depth (Table 7, Fig 9). Predictive utility of the model was fair. Randomization testing indicated low risk of incorporating a non-informative bathymetric variable.

**Fig 10:**
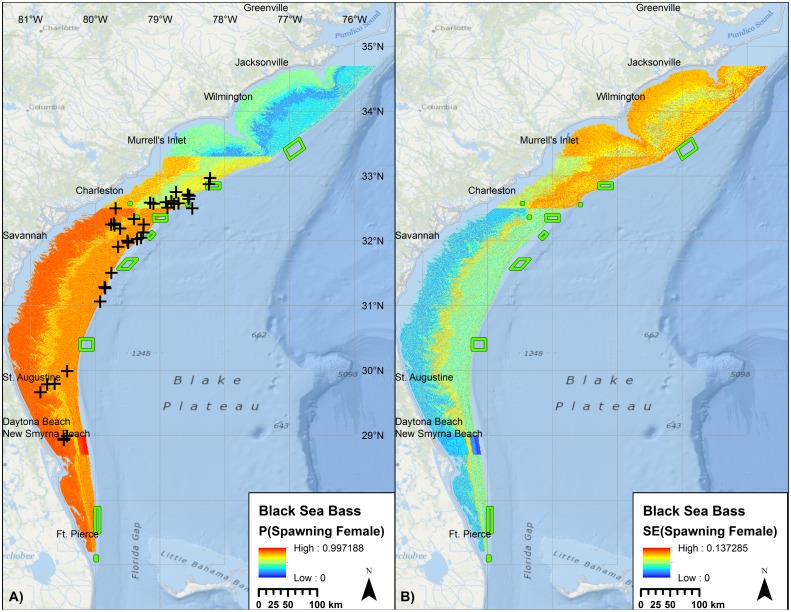
Probability of encountering a spawning condition female Black Sea Bass. Predicted mean (left) and standard error (right) probabilities of observing spawning condition female Black Sea Bass at time and conditions of peak spawning, relative to external validation observations (+). Raster color-coding based on 2.5 standard deviations from the mean. Green boxes denote no-take marine protected areas and SMZs. Basemap courtesy ESRI Ocean Basemap and partners.

For Black Sea Bass, 72% of the total variability in the presence of spawning condition females was explained by bottom water temperature, latitude, lunar phase, year, depth, maximum fine-scale BPI and mean curvature from the CRM bathymetry (Table 7, Fig 10). Based on AUC, the predictive utility of the model was excellent. The bathymetric variables were of limited explanatory value; the model had moderate risk of inclusion of a spurious bathymetric variable. Distinct gradients were observed in the predictive map due to quartile binning of latitude.

For Scamp throughout the SERFS sampling domain, 43% of the total variability in the presence of spawning condition females was explained by year, temperature, latitude, lunar phase, depth, and mean BPI from the CRM bathymetry (Table 7, S3 Fig). Based on AUC, the predictive utility of the model was good. Randomization testing indicated a relatively high risk of incorporating a non-informative bathymetric variable.

### External validation of predicted spawning locations

The predictive model for Gray Triggerfish was compared to MARMAP fishery-dependent collections (S1 Fig). The Z-scores for the probability of encountering a spawning condition female at the two SERFS sites that overlapped the predictive map were 2.6 and -0.2, respectively.

The predictive model for White Grunt was compared to MARMAP fishery-dependent collections (S2 Fig). The only fishery-dependent MARMAP sample located in this layer had a Z-score of 2.1.

**Fig 11:**
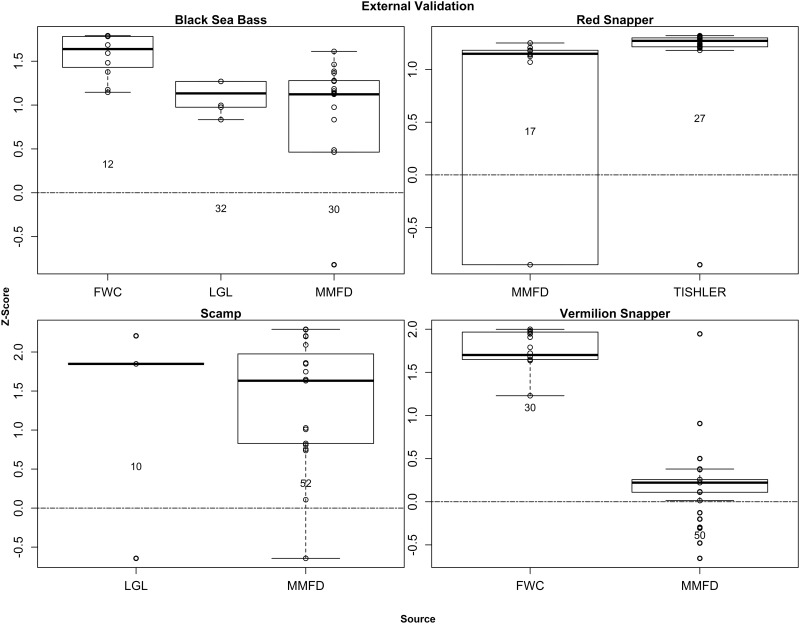
External validation of spawning predictions. Boxplots of model-predicted Z-score standardized probabilities of collecting a spawning female underlying locations where spawning females were collected by Florida Fish and Wildlife Conservation Commission (FWC; Lowerre-Barbieri et al. [39]), LGL Ecological Research Associates ([67]), MARMAP Fishery Dependent Sampling (MMFD), and anecdotal reports from fishers (‘Tishler’) collected by Tishler-Meadows [66]. Z-Scores above zero were interpreted as providing support for model predictions. Inset numbers denote sample sizes.

The predictive model for Red Snapper (Fig 7) was compared to MARMAP fishery-dependent collections and information collected from fishers [66]. Of the 44 external validation points, 38 (86%) were located at sites with predicted probabilities that exceeded the mean probability in the model domain (Fig 11).

The predictive models for Vermilion Snapper were compared to MARMAP fishery-dependent collections and FWC fishery-independent samples of spawning condition female Vermilion Snapper. Of the 80 external validation points in the broad-scale predictive layer for Vermilion Snapper (Fig 8), 69 (86%) were located at sites with predicted probabilities that exceeded the mean probability in the model domain (Fig 11). The Z-score for the only fishery-dependent MARMAP sample located in the fine-scale predictive layer was -0.4 (Fig 9).

The predictive model for Black Sea Bass (Fig 10) was compared to incidental catch of spawning condition female Black Sea Bass from the LGL sampling program, MARMAP fishery-dependent collections, and FWC fishery-independent samples. Of the 74 external validation points, 68 (92%) were located at sites with predicted probabilities that exceeded the mean probability in the model domain (Fig 11).

The predictive model for Scamp was compared to LGL and MARMAP fishery-dependent collections (S3 Fig). Of the 62 external validation points, 58 (94%) were located at sites with predicted probabilities that exceeded the mean probability in the model domain (Fig 11).

## Discussion

For most species evaluated, our methods were able to elucidate the spatial, temporal, and environmental cues to peak spawning. Several multispecies spawning areas were observed, and most species appeared to utilize spawning areas across many years. Logistic regression approaches generated spatial predictions for the probability of encountering spawning condition females. Internal cross-validation indicated these models had fair to excellent predictive utility, depending on the species. Independent validation provided a strong indication that the predictive models would not frequently deliver false negatives.

### Balistidae

Gray Triggerfish are gonochoristic, small-bodied [size at maturity = 32.8 cm [68]] fish [53,69]. Consistent with other members of the family Balistidae [70], Gray Triggerfish are reported to form haremic groups [71], with male Gray Triggerfish establishing territories with nests in the sediment either in or around reef structure [53,69]; parents release demersal gametes and protect the nest for 24–48 h after which time the larvae enter a pelagic stage [69]. Spawning Gray Triggerfish were collected at numerous, broadly distributed locations along and just inshore of the shelf-edge. Given their unique reproductive strategy, it is unsurprising that they were less commonly observed at multispecies spawning areas than other stocks. Model fits for Gray Triggerfish were good, but explained a relatively low percentage of the overall variability (34%). Peak spawning months were identified as June-July, which agrees with previously published studies (Table 6). Peak spawning was predicted around the new moon at flat locations in the 49–78 m depth range. The timing of spawning around the new moon may reduce nocturnal predation on demersal eggs, although this was not explicitly testable using the data at hand. Spawning aggregations for other members of the family Balistidae have been observed forming just prior to the new moon, full moon, or both (*Balistoides viridescens* Donaldson and Dimalanta [72], *Canthidermis sufflamen* Heyman and Kjerfve [38]).

### Haemulidae

White Grunt are small-bodied (size at maturity = 16.7 cm [73]), gonochoristic and have previously been reported to reproduce throughout the year in different areas of the Caribbean, with possible multiple spawning peaks [74,75]. Peak spawning was predicted inshore of the shelf-edge (31–37 m depth) in 24.7–26.2°C waters during the waxing half moon in May and June. Studies in other regions have observed peak spawning for White Grunt during months corresponding to the lowest regional water temperatures [74,76,77], but this was not observed in the SERFS database, possibly due to limited winter sampling. More recently, Stallings et al. [78] found strong evidence for a peak in hydrated eggs in female White Grunt in the eastern Gulf of Mexico around the full moon in April. It is unsurprising that peak spawning differs between regions, as fish evolve different strategies to maximize survival of their offspring in different locations [79]. Peak lunar phase and peak month of spawning can vary by species across relatively small scales [35].

### Lutjanidae

Red Snapper are moderately-sized (size at maturity = 37.1 cm [80]) gonochoristic fish and are reported to spawn May-October, with a peak June-September [81]. Little is known about Red Snapper spawning ecology in the SEUS; however, an aggregation of 20–30 Red Snapper that “appeared to be a spawning aggregation” was observed via submarine at Scamp Ridge off South Carolina in August 2002 [54]. Red Snapper spawning condition females were encountered in limited numbers by SERFS despite sampling during their spawning season. Their core distribution appears to be centered off Northeast Florida, an area of historically low SERFS sampling. More recent sampling targeting Red Snapper provided additional statistical power to the Red Snapper model fits. Unfortunately, insufficient samples were available in the MB bathymetry to fully evaluate the impacts of biogeomorphology on spawning for this stock. However, it appeared that Red Snapper peak spawning takes place in 24.7–29°C waters prior to the new moon at locations with high bathymetric curvature in the 24–30 m depth range off Northeast Florida during June and July. The predictive model confirms SEUS observations that Red Snapper spawn at numerous protruding hardbottom locations [39] and is supported by findings in the Gulf of Mexico that Red Snapper spawn on rocky ridges and relatively steep delta terrace drop-offs [51].

Vermilion Snapper are gonochoristic, small-bodied [size at maturity = 15.0 cm [82]] fish previously reported to spawn approximately every five days or about 35 times a year between April and September [83]. The large size of multi-year spawning areas for this species suggests a relatively broad use of habitat for spawning rather than isolated sites. A fair model fit in the high-resolution bathymetry indicated that female Vermilion Snapper are most likely to be found in spawning condition in 20.5–21.6°C waters at deep (52–63 m) high-profile ridges prior to the new moon in August. It is noteworthy that Vermilion Snapper was the only species with a large sample size (n = 179) in the MB bathymetry layer, and our findings for their association with high-profile habitats were consistent with observations for tropical reef fish species [23,25]. By contrast, the same analysis applied to the broad-scale bathymetry found higher probability of encountering spawning condition females in areas of no slope. These contradictory findings suggest that results are highly dependent on spatial scale of the analysis, a problem which abounds in the marine realm [84].

### Serranidae

Black Sea Bass are moderately-sized (female length at maturity = 13.5 cm [85]) protogynous hermaphrodites that undergo sex transition between ages 1 to 8 years [86]. Not all latitude zones were represented during most peak spawning months; thus, the model-reported differences in probability of spawning at different latitudes may be somewhat misleading. Despite limited sampling in the winter, the Black Sea Bass model predicted peak spawning at moderately curving high-profile ridges in 10.4–20.5°C inshore waters (13–22 m depth) during the full moon, which aligns well with previous studies on the species (see Table 6). This suggests that spawning activity by location may not vary significantly between peak and non-peak spawning times for this stock.

Scamp are large-bodied [female size at maturity = 35.3 cm [87]] protogynous hermaphrodites known to aggregate in deep water (>40 m) to spawn at high-relief sites on the shelf-edge, with or without a drop-off [15,50,51]. Peak spawning was predicted near the new moon in 19.7–21.6°C waters on the shelf edge (48–51 m) at high-profile ridges off South Carolina. Previous studies have reported Scamp probably spawn during the late afternoon and evening, with a peak during March-May around the new moon and full moon [87]. Scamp have been reported in gray-head courtship color phase [88] at Georgetown Hole and Julians Ridge off South Carolina and at Sebastian Pinnacles, Jacksonville Scarp, and St. Augustine Scarp off Florida [67,89,90].

Snowy Grouper are large-bodied (female size at maturity = 54.1 cm [55]) protogynous hermaphrodites [55]. Adults are generally associated with the upper continental slope (>75 m) in habitats characterized by rocky ledges, cliffs, and swift currents [91,92]. Snowy Grouper were also often observed by SERFS video sampling in low relief rocky outcrops and pavement habitats, often mixed in with Blueline Tilefish (N. Bacheler, SEFSC-SEFIS, pers. comm. 2016). We observed spawning condition female Snowy Grouper at depths from 175–223 m. Multi-year use of spawning areas identified at least three areas offshore and south of the existing Northern South Carolina MPA (see Fig 3).

### Applications to management

Because traditional management efforts in the SEUS have been insufficient to stop overfishing for several species, such as Red Snapper, Warsaw Grouper and Speckled Hind, other management tools are being explored—particularly spatial tools designed to reduce bycatch mortality and protect spawning fish. There is a growing interest in the SEUS to develop management tools to protect spawning fish, as evidenced by the Council’s development of a System Management Plan to monitor new and existing no-take areas and their approval of Amendment 36 to the Snapper-Grouper Fishery Management Plan, which develops no-take areas, referred to as ‘Spawning Special Management Zones’ (SMZs) to protect important spawning fish and associated habitats [6]. Our analysis contributed to management decision-making for this amendment and identifies other areas that may merit spatial protection.

Not all large snapper and grouper species spawn in highly predictable, site-specific locations. Species such as Red Snapper, Gray Snapper (*Lutjanus griseus*), and Yellowtail Snapper (*Ocyurus chrysurus*) and species that feed lower on the food chain (e.g. scarids, labrids, acanthurids) or that achieve smaller maximum size may spawn repeatedly at predictable locations; however these spawning events may be broadly-distributed in space and time, with a relatively low fraction of the spawning population represented at any given event [8,12,39]. These stocks may benefit more from seasonal protections during peak spawning as opposed to spatial closures [8,11]. Our analysis suggests that there are broadly distributed spawning areas for Gray Triggerfish, White Grunt, Red Snapper, Vermilion Snapper, and Black Sea Bass, although we note that the broad spatial patterns in the predictive maps are partly an artifact of the level of binning used in our analysis. Spatial management tools focused specifically on spawning protection for these species, such as those employed for large-bodied serranids in other regions [93,94]; would likely prove unsuccessful unless they were on a correspondingly large spatio-temporal scale—at least, until data become available that would allow for more high-resolution analysis and prediction of spawning activity.

An understanding of spawning ecology and reproductive resilience is critical to the effective management of exploited fish stocks [3]. An important first step towards incorporation of reproductive resilience into stock assessments is quantifying spawning site diversity and density [3]. We identified numerous multi-annual spawning areas; however, the resolution of our data were insufficient to conclude whether these areas contained transient spawning aggregations, simple migratory spawning, or resident spawning. Several species are known to spawn in aggregations in the SEUS. However, with the exception of Gag [95,96], most of these species occur well south of the area of focus for this study [i.e., Black Grouper [97], Gray Snapper [98], and Mutton Snapper [20]]. Due to reduced populations following decades of fishing pressure, limited winter sampling, and limited sampling in southern Florida, collections of spawning condition females for these species were limited. Based on their repeated use of numerous, broadly-distributed spawning locations, we anticipate a type of simple migratory or, potentially, resident or near-resident spawning behavior in a vast number of locations over a broad geographic range for Gray Triggerfish, White Grunt, Red Snapper, Vermilion Snapper, and Black Sea Bass. The presence of these species in an SMZ may indicate some value in closing the area; however, their spawning presence does not, by itself, fully support closure of that area. White Grunt, Red Snapper, and Vermilion Snapper were common at multispecies spawning areas, and may be a good indicator of favorable spawning habitats for some non-aggregating species.

Our analysis suggested that immediate improvements in spawning protection for the two larger-bodied serranids (Scamp and Snowy Grouper) might be possible. Several multi-year spawning areas for these species were identified offshore of the boundaries of existing MPAs. The reorientation or extension of these protected areas could provide useful protection to the spawning stock. As protogynous hermaphrodites, Scamp and Snowy Grouper are particularly vulnerable to sperm limitation [15,51,55]. Snowy Grouper are currently considered overfished by the National Marine Fisheries Service based on a 2013 assessment [42]. Recent reviews of Scamp have called for close monitoring of the population due to a decreased percentage of males in the population and a loss of older, larger females [87]. Additionally, the periods of peak spawning identified in Table 6 suggest that although the Council’s January-April closure to grouper harvest may provide protection for spawning Gag and Red Grouper, it is mistimed for spawning Scamp, Snowy Grouper, Speckled Hind, and Warsaw Grouper.

Intensive fishing on natural spawning aggregations may alter their dynamics. It is well-documented that overexploitation results in smaller, faster-maturing fish [99]. Additionally, if fish spawning at aggregations are disproportionately targeted relative to members of the population spawning outside of aggregations, fishing may act to select for non-aggregating spawners. Fishers have indicated there were several known large Red Snapper spawning aggregation sites in the 1960s and 1970s off Florida that no longer form [44], and more recent evidence suggests Red Snapper spawn in small groups in a broad array of habitats [39,66]. In the context of aggregation overfishing, low density spawning groups at a diversity of locations may confer reproductive resilience [3,39].

To date, no spawning aggregations have been formally documented by researchers in the Council’s jurisdiction north of Jupiter Inlet, Florida. There are several purported spawning aggregation or simple migratory spawning sites from east-central Florida northward, and a growing body of indirect evidence for their occurrence in various locations [5,66]. Georgetown Hole (or Devil’s Hole) is perhaps the most obvious example of a multispecies spawning area that has been identified by multiple fishers [5]. Concurrent with the data mining efforts presented above, several of the authors of this paper have also been piloting a collaborative protocol for documenting spawning areas in the field [48]. Based on observations of two or more female fish in spawning condition, spawning in the Georgetown Hole area was confirmed for Scamp, Snowy Grouper, and Blueline Tilefish [67]. Based on single samples of female fish in spawning condition, Greater Amberjack, Mutton Snapper (*Lutjanus analis*), Yellowedge Grouper *(Epinephelus flavolimbatus)*, and Warsaw Grouper were also confirmed in spawning condition in the Georgetown Hole, SC area [67]. The degree to which this information reflects species-level aggregation spawning, simple migratory spawning, or other types of spawning is under further evaluation. The Council recently approved a 7.85 km^2^ SMZ at Georgetown Hole [6]. Based on the sizes of multi-year spawning locations identified in this study (see Table 5 and Fig 3), if properly placed and configured, this SMZ might be sufficient to protect spawning locations for a variety of species.

### Data limitations

This study was completed in large part from data mining the multi-decadal SERFS database. Although this comprehensive fishery-independent survey represents the longest-standing, most consistent sampling approach in the SEUS, the fishery-independent component of SERFS was not specifically designed to evaluate timing and location of spawning activity. However, it has proven useful for this purpose. The SERFS and FWC fishery-independent data contained limited information on larger, longer-lived species of groupers and snappers in the SEUS (e.g. Warsaw Grouper, Speckled Hind, Gag, Scamp, Snowy Grouper, Yellowedge Grouper *Epinephelus flavolimbatus*, and Cubera Snapper, *Lutjanus cyanopterus*) due to limited sampling in the southern Florida region and limited sampling during the winter months that represent the spawning season for most grouper species. These large-bodied species may follow similar spatial and temporal spawning patterns of more tropical and insular species (i.e. large, infrequent, transient aggregations at distinctive geomorphological features), but care should be taken not to infer that all distinctive geomorphological features host spawning aggregations.

An absence of sampling does not imply an absence of spawning activity—there are many areas and months that remain poorly sampled. Not surprisingly, the predictive utility of our models and our observations of multi-year and multispecies spawning areas were clustered in the region of highest sampling (e.g. South Carolina). Additionally, the primary sampling gear utilized by SERFS is the chevron trap; due to difficulty with recovery, traps are seldom set on the deep, rocky ledges in high current zones where reef fish spawning activity has been documented in other regions [23]. Despite this limitation, several associations between high-profile ridges and high probability of encountering spawning condition females were noted. We had hoped to utilize the SEAMAP 1199 habitat grid [100] as a predictor of spawning habitats. Unfortunately, the spatial coverage of this layer was too limited to be useful in a statistical modeling framework and the SERFS samples were predominantly constricted to identified hardbottom habitats, reducing the possible influence of this covariate (see Fig 9).

### Future improvements

Our understanding of spawning ecology in the SEUS would be greatly enhanced by an expansion of fishery-independent sampling into southern Florida and into the winter months. Additionally, increased sampling on high-relief, high current habitats using gears other than the chevron trap might increase encounters with large-bodied aggregating spawners. Our ability to distinguish bathymetric features that may be aggregation sites would be greatly enhanced by more comprehensive fine-scale multibeam sampling of the region. This study has identified limited availability of high-resolution bathymetry data within the SEUS. The CRM provides comprehensive bathymetric information, but at a very low resolution and with known errors and accuracy issues (NAF, unpublished data). Comprehensive high resolution bathymetric data would allow much more fine-scale analysis of the effects of geomorphology on spawning site distribution by species. Improved bathymetric data would allow a re-analysis of this database at spatial scales that might be more meaningful to spawning reef fish, a key factor in predictive ability of such models [84].

Many studies have documented shifting distributions in marine species in response to climate change [101–103]. For the six species from four reef fish families tested, temperature and/or latitude had significant effects on the probability of collecting spawning condition females. Therefore, the spawning activities of these species may be particularly influenced by climate change. The spawning season of these stocks and other stocks protected by seasonal spawning closures (e.g., shallow-water groupers) should be tracked through time relative to environmental factors, to ensure alignment of management objectives with the reproductive ecology of the stock. The productivity of stocks with peak spawning predicted in cooler waters (i.e., Black Sea Bass, Scamp, Vermilion Snapper) may be particularly vulnerable to climate change. A significant interaction between latitude and month was detected for Vermilion Snapper, suggesting there may be some plasticity for dealing with climate change for this species.

As discussed above, this study has revealed gaps in the data needed to fully characterize spawning areas and times by species in the SEUS. We offer the following suggestions for improved data collection, recognizing that many will be subject to funding constraints:

1. Conduct region-wide histological sampling, especially in southeastern Florida (south of 27° N) where aggregations of Gray Snapper (NAF, pers. obs., 2014), Mutton Snapper [20], Warsaw Grouper (D. DeMaria, pers. comm., 2016), Black Grouper, Red Grouper, and Red Hind *Epinephelus guttatus* [104] have been reported.
2. Increase winter sampling to provide a better understanding of the spawning dynamics for most groupers and many other species (see Table 6).
3. Increase sampling at high-relief, high current locations where multispecies aggregations are probable [23], using hook-and-line or longline gears to avoid trap loss.
4. Evaluate histological samples to ascertain whether aggregating males demonstrate a more discrete spawning season than resident males.
5. Use cooperative research with commercial fishermen to collect video and biological samples for under-represented species/areas/times similar to approaches outlined in Heyman [48].
6. Integrate these monitoring approaches into the Council’s MPA System Management Plan (http://safmc.net/SystemManagementPlan) as a required monitoring protocol for existing and newly implemented MPAs and SMZs.

### Conclusion

Despite the various data limitations discussed above, timing and location of spawning was identified or confirmed for numerous commercially-important reef fish species in the SEUS, at broad spatial scales. Many of the evaluated species appeared to spawn at myriad locations along the shelf-edge or inshore. Although many reef fishes important in commercial and recreational fisheries off the SEUS spawn across broad shelf areas, it is evident that some spawning is localized. Often, local spawning grounds were utilized by several species. Many multi-year and multispecies spawning locations were located close to existing MPAs, where expansion or reorientation of those MPAs might provide conservation benefits. For example, spawning condition female Vermilion Snapper and Scamp have been repeatedly collected on a ledge just north of Edisto MPA and these locations could be contained by a reorientation of this MPA (see Fig 3).

For those species that form aggregations, cooperative research approaches and expanded SERFS sampling might help elucidate their spawning locations. Unfortunately, intensive fishing over the past several decades may have reduced these aggregations to the point they might not be recognizable as such. In the Gulf of Mexico, the Madison-Swanson and Steamboat Lumps Marine Reserves demonstrated remarkable recoveries of spawning aggregations of Gag and Scamp after nearly a decade of protection [51]. At Riley’s Hump near the Dry Tortugas islands in southwestern Florida, a nearly extirpated Mutton Snapper spawning aggregation has become a regional success story after over a decade of protection, and likely contains spawning aggregations of other snapper and grouper species [20,104]. These locations were closed based on shelf geomorphology and information from fishers indicating known historical reef fish spawning activity at the sites [105]. The SEUS contains many similar sites, some with fishery-independent documented spawning activity of commercially-important species. Spawning site closures for species that are appropriately sized and enforced to reduce fishing pressure on spawning fish may improve the sustainability of regional stocks through increased recruitment. These closures should be left in place and monitored for sufficient time relative to the lifespan of the species to allow for a meaningful recovery. Sufficient monitoring to generate a time series of the abundance of spawning individuals would allow an empirical demonstration of the utility of the closure and would also promote more effective management [106]. Expanding these approaches to other regions is recommended [107], as spawning migrations and larval distribution patterns may cross regional and national boundaries.

## Supporting information

S1 Table Supporting references for Table 6 spawning seasons. (DOCX)

S1 File Plots of regression model parameter estimates from Table 7. (DOCX)

S2 File Three-dimensional bathymetric video of southeastern United States indicating locations of fishery-independent collections of spawning condition female reef fish. (MP4)

S1 Fig Probability of encountering a spawning condition female Gray Triggerfish. Predicted mean (left) and standard error (right) probabilities of observing spawning condition female at time and conditions of peak spawning, relative to external validation collections (+). Raster color-coding based on 1.5 standard deviations from the mean. Green boxes indicate no-take marine protected areas.
(TIF)

S2 Fig Probability of encountering a spawning condition female Scamp. Predicted mean (left) and standard error (right) probabilities of observing spawning condition female at time and conditions of peak spawning, relative to external validation collections (+). Raster color-coding based on 1.5 standard deviations from the mean. Green boxes indicate no-take marine protected areas.
(TIF)

S3 Fig Probability of encountering a spawning condition female White Grunt. Predicted mean (left) and standard error (right) probabilities of observing spawning condition female at time and conditions of peak spawning, relative to external validation collections (+). Raster color-coding based on 1.5 standard deviations from the mean. Green boxes indicate no-take marine protected areas.
(TIF)
