# Supplementary material for: Timing and locations of reef fish spawning off the southeastern United States
Source: PLoS One. 2017 Mar 6;12(3):e0172968. doi: 10.1371/journal.pone.0172968 (PMC5338871; doi:10.1371/journal.pone.0172968)
Supplement: S1 Table — (DOCX) [file pone.0172968.s001.docx]

**Table S1.** Spawning timing references from Table 6.

| **#** | **Citation** |
| --- | --- |
| 1 | Burgos JM, Sedberry GR, Wyanski DM, Harris PJ (2007) Life history of red grouper (*Epinephelus morio*) off the coasts of North Carolina and South Carolina. Bulletin of Marine Science 80(1):45-65. |
| 2 | Cuellar N, Sedberry GR, Machowski DJ, Collins MR (1996) Species composition, distribution and trends in abundance of snappers of the southeastern USA, based on fishery independent sampling. In: Arreguin-Sanchez F, Munro JL, Balgos MC, Pauly D (eds) Biology, fisheries and culture of tropical groupers and snappers. ICLARM Conference Proceedings 48, pp 59–73 |
| 3 | Daniel EA (2003) Sexual maturity, spawning dynamics, and fecundity of Red Porgy, *Pagrus pagrus*, off the southeastern United States. M.S. Thesis, College of Charleston, 79 p |
| 4 | Erickson DL, Harris MJ, Grossman GD (1985) Ovarian cycling of Tilefish (*Lopholatilus chamaeleonticeps*, Goode and Bean) from the South Atlantic Bight, U.S.A. Journal of Fish Biology 27:131–146 |
| 5 | Gilmore RG, Jones RS (1992) Color variation and associated behavior in the epinepheline groupers, *Mycteroperca microlepis* (Goode and Bean), and M. phenax (Jordan and Swain). Bulletin of Marine Science 51:83–103 |
| 6 | Harris PJ, Wyanski DM, Mikell PTP (2004) Age, growth, and reproductive biology of Blueline Tilefish along the southeastern coast of the United States, 1982–1999. Transactions of the American Fisheries Society 133:1190–1204 |
| 7 | Harris PJ, Wyanski DM, White DB, Mikell PP, Eyo PB (2007) Age, growth, and reproduction of Greater Amberjack off the southeastern U.S. Atlantic coast. Transactions of the American Fisheries Society 136:1534–1545 |
| 8 | Harris PJ, Wyanski DM, White DB, Moore JL (2002) Age, growth, and reproduction of Scamp, *Mycteroperca phenax*, in the southwestern North Atlantic, 1979-1997. Bulletin of Marine Science 70(1):113–132 |
| 9 | Hood PB, Johnson AK (1999) Age, growth, mortality, and reproduction of Vermilion Snapper, *Rhomboplites aurorubens*, from the eastern Gulf of Mexico. Fishery Bulletin 97(4):828–841 |
| 10 | Kelly A (2014) Age, growth, and reproduction of Gray Triggerfish *Balistes capriscus* off the southeastern U.S. Atlantic coast. M.S. Thesis, College of Charleston, 54 p |
| 11 | LGL Ecological Research Associates Inc. (LGL ERA) (2015) Cooperative prediction and verification of a multi-species spawning site at Georgetown Hole: Summary of results from 2014. South Atlantic Fishery Management Council, Charleston, SC, 9 pp. |
| 12 | Matheson RH III, Huntsman GR, Manooch CS III (1986) Age, growth, mortality, food and reproduction of the Scamp, *Mycteroperca phenax*, collected off North Carolina and South Carolina. Bulletin of Marine Science 38:300–312 |
| 13 | McGovern JC, Wyanski DM, Pashuk O, Manooch CS III, Sedberry GR (1998) Changes in the sex ratio of Gag, *Mycteroperca microlepis*, from the Atlantic coast of the southeastern United States during 1976–1995. Fishery Bulletin U.S. 96:797–807 |
| 14 | Padgett SM (1997) Age, growth and reproductive biology of the White Grunt, *Haemulon plumieri*, along the southeast Atlantic coast of the United States. M.S. Thesis, College of Charleston, 61 p |
| 15 | SEDAR (2011) SEDAR 25 - South Atlantic Black Sea Bass stock assessment report. SEDAR, North Charleston, 146 p, available online at http://www.sefsc.noaa.gov/sedar/Sedar_Workshops.jsp?WorkshopNum=25 |
| 16 | SEDAR (2013) SEDAR 36 – South Atlantic Snowy Grouper stock assessment report. SEDAR, North Charleston, 146 p [http://www.sefsc.noaa.gov/sedar/Sedar_Workshops.jsp?WorkshopNum=36] |
| 17 | Sedberry GR, Pashuk O, Wyanski DM, Stephen JA, Weinbach P (2006) Spawning locations for Atlantic reef fishes off the southeastern U.S. Proceedings of the Gulf and Caribbean Fisheries Institute 57:463–514 |
| 18 | White DB, Palmer SM (2004) Age, growth, and reproduction of the Red Snapper, *Lutjanus campechanus*, from the Atlantic waters of the southeastern U.S. Bulletin of Marine Science 75:335–360 |
| 19 | Wyanski DM, White DB, Barans CA (2000) Growth, population age structure, and aspects of the reproductive biology of Snowy Grouper, *Epinephelus niveatus*, off North Carolina and South Carolina. Fishery Bulletin 90(1):198-218 |
| 20 | Ziskin GL, Harris PJ, Wyanski DM, Reichert MJ (2011) Indications of continued overexploitation of speckled hind along the Atlantic Coast of the southeastern United States. Transactions of the American Fisheries Society 140(2):384-98. |
