# Supplementary material for: Timing and locations of reef fish spawning off the southeastern United States
Source: PLoS One. 2017 Mar 6;12(3):e0172968. doi: 10.1371/journal.pone.0172968 (PMC5338871; doi:10.1371/journal.pone.0172968)
Supplement: S1 File — (DOCX) [file pone.0172968.s002.docx]

**Black sea bass**

**

**


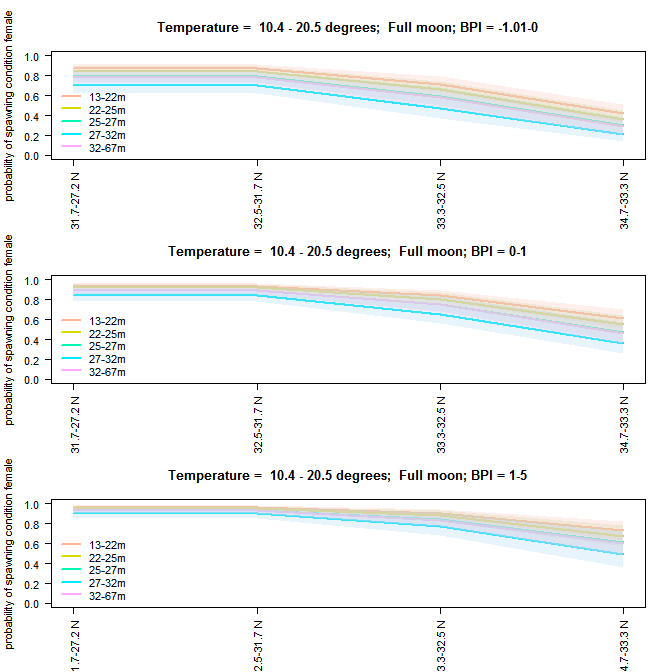


**Gray triggerfish**

**

**

**Red snapper**

**

**

**Scamp**

**

**

**Vermilion snapper (broad-scale)**

**

**

**Vermilion snapper (fine-scale, SC only)**

**

**

**White grunt**

**

**
