# Supplementary figures and images for: Timing and locations of reef fish spawning off the southeastern United States
Source: PLoS One. 2017 Mar 6;12(3):e0172968. doi: 10.1371/journal.pone.0172968 (PMC5338871; doi:10.1371/journal.pone.0172968)

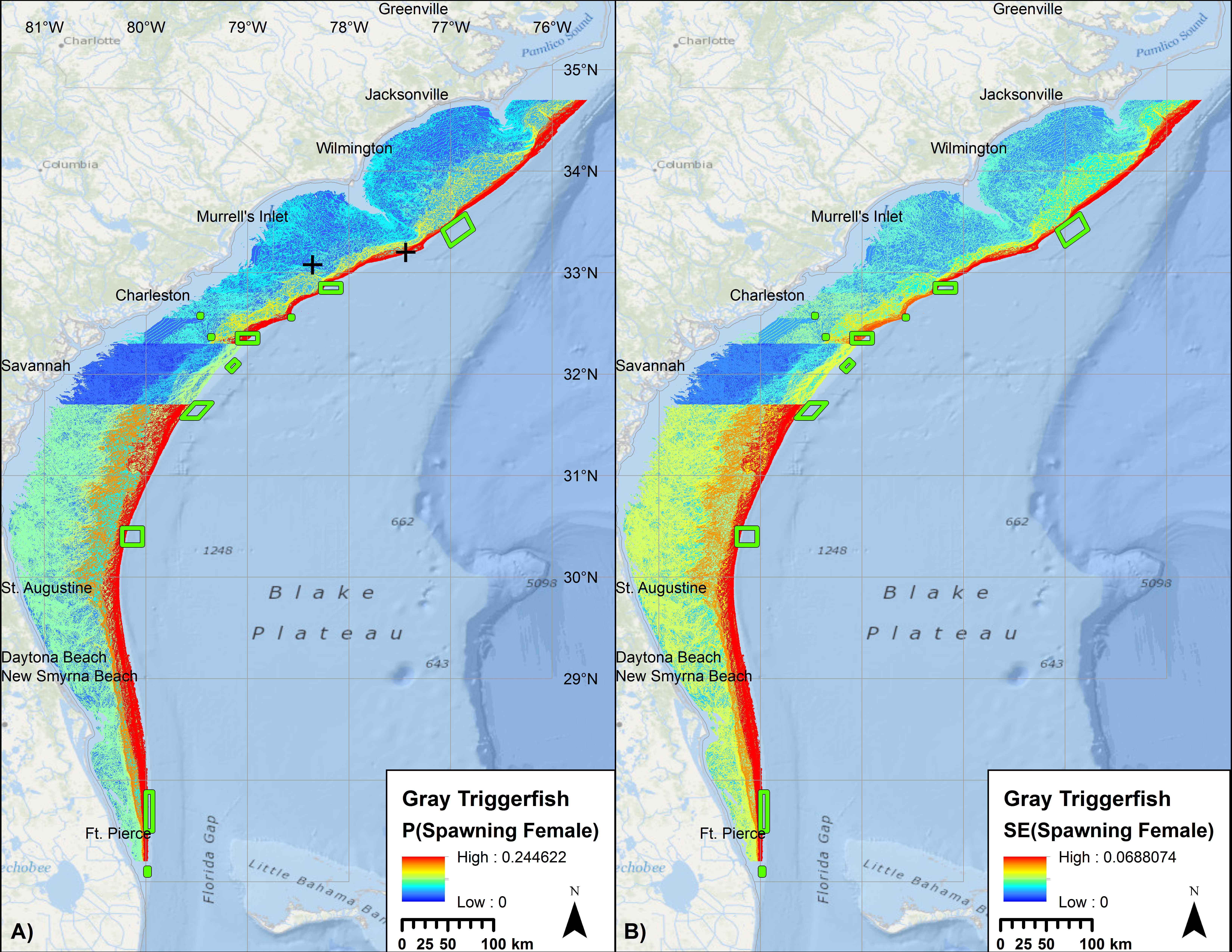

Supplement: S1 Fig — Predicted mean (left) and standard error (right) probabilities of observing spawning condition female at time and conditions of peak spawning, relative to external validation collections (+). Raster color-coding based on 1.5 standard deviations from the mean. Green boxes indicate no-take marine protected areas. (TIF) [file pone.0172968.s004.tif]

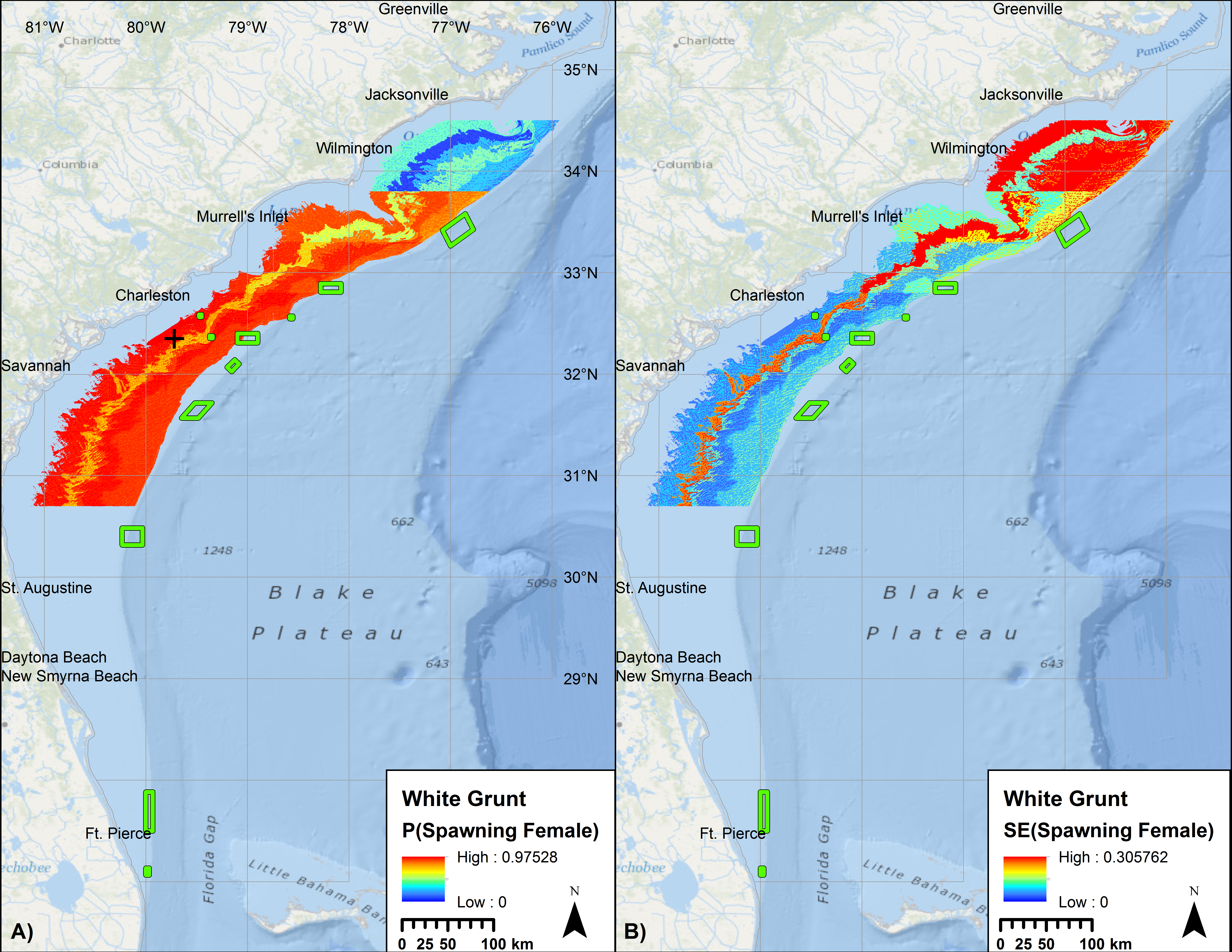

Supplement: S2 Fig — Predicted mean (left) and standard error (right) probabilities of observing spawning condition female at time and conditions of peak spawning, relative to external validation collections (+). Raster color-coding based on 1.5 standard deviations from the mean. Green boxes indicate no-take marine protected areas. (TIF) [file pone.0172968.s005.tif]

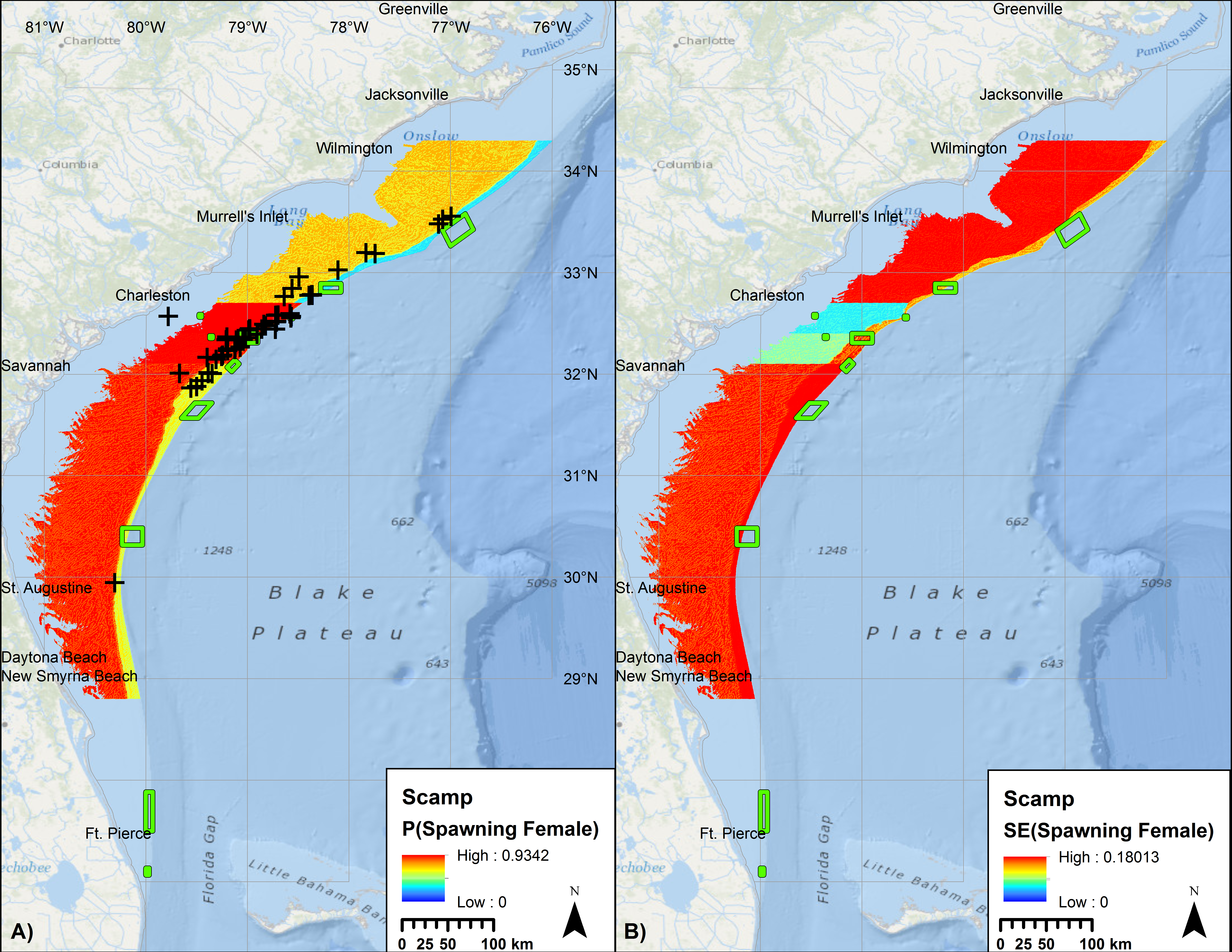

Supplement: S3 Fig — Predicted mean (left) and standard error (right) probabilities of observing spawning condition female at time and conditions of peak spawning, relative to external validation collections (+). Raster color-coding based on 1.5 standard deviations from the mean. Green boxes indicate no-take marine protected areas. (TIF) [file pone.0172968.s006.tif]
